# Yield of Whole Genome Sequencing for Pathogenic Single Nucleotide Variants in Congenital Heart Disease: A Systematic Review and Meta‐Analysis

**DOI:** 10.1002/pd.6878

**Published:** 2025-09-04

**Authors:** Hiba J. Mustafa, Parisa Najjariasl, Faezeh Aghajani, Enaja V. Sambatur, Andrew Rodenbarger, Stephanie Guseh, Amy E. Roberts, Alireza A. Shamshirsaz

**Affiliations:** ^1^ Division of Maternal‐Fetal Medicine The Fetal Center at Riley Children's and Indiana University Health Indiana University School of Medicine Riley Children's Hospital Indianapolis Indiana USA; ^2^ Shahid Beheshti University of Medical Sciences Tehran Iran; ^3^ Fetal Care and Surgery Center Division of Fetal Medicine and Surgery Boston Children's Hospital Harvard Medical School Boston Massachusetts USA; ^4^ Riley Heart Center Division of Pediatric Cardiology Indiana University School of Medicine Riley Children's Hospital Indianapolis Indiana USA; ^5^ Division of Maternal‐Fetal‐Medicine Brigham and Women's Hospital Harvard Medical School Boston Massachusetts USA; ^6^ Division of Genetics and Genomics Boston Children's Hospital Boston Massachusetts USA; ^7^ Department of Cardiology Boston Children's Hospital Boston Massachusetts USA

## Abstract

**Objective:**

This systematic review and meta‐analysis aimed to assess the diagnostic yield of pathogenic or likely pathogenic (P/LP) single nucleotide variants (SNVs) using whole genome sequencing (WGS) in congenital heart disease (CHD).

**Methods:**

A systematic search of three databases (2000–2024) was conducted, and two reviewers independently screened studies and extracted data following PRISMA and MOOSE guidelines. Pooled proportions were calculated using a random‐effects model, and study quality was assessed using modified STARD criteria.

**Results:**

Fourteen studies were included, comprising 933 CHD cases, of which 165 had P/LP SNVs. The overall diagnostic yield of WGS for P/LP SNVs was 17.83%, with a yield of 9.83% in isolated CHD cases (without other abnormalities) and 22.36% in syndromic cases (with extracardiac anomalies, developmental abnormalities, or distinctive features). Among 105 cases from four studies with negative chromosomal microarray (CMA) results, 20 had subsequently positive findings by WGS, yielding a 20% incremental diagnostic benefit of WGS over CMA.

**Conclusions:**

These findings highlight the utility of WGS in identifying clinically relevant SNVs in CHD and suggest that WGS should be considered in the diagnostic workup of CHD, particularly in syndromic cases, to guide personalized management and multidisciplinary care.

**PROSPERO Registration:**

CRD42025634370.

## Summary

- What is already known?
  ◦ CHD is a common birth defect with genetic causes. The diagnostic yield of WGS in CHD has not been systematically evaluated.
- What does this study add?
  ◦ The pooled diagnostic yield of WGS in CHD is 17.8%, increasing to 22.4% in syndromic cases.
  ◦ In isolated CHD, the WGS yield drops to 9.8%. These findings support a tiered approach in isolated cases and earlier utilization of WGS in syndromic cases.

## 1 Introduction

Congenital heart disease (CHD) is the most prevalent congenital disorder, with an incidence rate ranging from 1.2 to 17 per 1000 live births, depending on the region [1]. In 2017, approximately 30 out of every 100,000 live births in the United States resulted in death due to CHD [2].

CHD has a complex etiology involving genetic, epigenetic, and environmental factors [3, 4, 5, 6]. Next‐generation sequencing (NGS) has revealed many of the genetic mechanisms of CHD [7, 8]. Genetic diagnosis enables accurate prenatal counseling, risk assessment, and informed reproductive planning [9]. Patients with a known genetic diagnosis, especially syndromic cases, benefit from individualized management and early screening for associated conditions [10, 11].

Genetic tests such as fluorescence in situ hybridization (FISH), karyotype, and chromosomal microarray array (CMA) have been offered as first‐line tests to detect chromosomal abnormalities in CHD patients. Since chromosomal anomalies vary across different types of CHD, the diagnostic yield of CMA can differ accordingly [12, 13].

CMA detects copy number variations (CNVs), which are large‐scale genomic duplications and deletions, but is limited in identifying SNVs or small insertions and deletions [14, 15]. These smaller‐scale mutations form the basis of monogenic disease and also play a major role toward structural abnormalities. SNVs can disrupt protein‐coding regions or crucial regulatory regions and lead to pathogenic changes that would otherwise not be detected through CNV analysis. Therefore, SNV detection is essential to achieve a comprehensive genetic diagnosis [16].

NGS overcomes these limitations by sequencing the entire genome or exome comprehensively [17]. Whole exome sequencing (WES) targets only protein‐coding regions (2.94% of the genome), whereas whole genome sequencing (WGS) covers both coding and non‐coding regions [18, 19]. Both technologies offer the capacity to detect large chromosomal deletions or duplications, small insertions or deletions, and single nucleotide variants in a single platform. The contribution of intronic regions to the diagnostic yield of WGS cannot be overlooked because these regions contain promoters and enhancers that regulate gene expression. Mutations within these regions may interfere with gene functions and drive CHD pathogenesis [20].

WGS is a highly useful tool, particularly when other methods are inconclusive [21]. In this systematic review, we evaluate the diagnostic capacity of WGS in identifying pathogenic and likely pathogenic single nucleotide variants in well‐established CHD genes.

## 2 Methods

This systematic review followed the *Cochrane Handbook for Systematic Reviews of Diagnostic Test Accuracy* [22] and adhered to PRISMA guidelines [23]. The study protocol was registered in PROSPERO (Code: CRD42025634370).

### 2.1 Search Strategy

A systematic search was conducted in four electronic databases: MEDLINE, Scopus, Web of Science, and Cochrane Library, in August 2024. The search strategy combined relevant Medical Subject Headings (MeSH) terms and keywords: (“Whole Genome Sequencing” AND (“Whole Exome Sequencing” OR “Chromosomal Microarray Analysis”)) AND (“Heart” AND (“anomal” OR “abnormal” OR “defect” OR “disease” OR “malform” OR “deform”)) AND (“congenital” OR “fetal” OR “birth”). Detailed search strategies are available in the Supporting Information S1. Reference lists and relevant reviews were manually checked for additional papers.

### 2.2 Selection of Studies

Studies generated from the search process were transferred into Rayyan^,^ a systematic review screening platform. Duplicates were identified in Rayyan and manually verified. The studies were selected in two phases. First, the titles and abstracts of the articles were independently screened by two reviewers (P.N. and E.S). Full‐text copies of the selected articles were independently assessed for their eligibility by the same two reviewers according to the inclusion and exclusion criteria described below. Disagreements between the two reviewers were resolved by discussion or by a third reviewer (H.M.). In the case of overlapping studies, only the largest and most complete data set was included.

### 2.3 Eligibility Criteria

Eligibility criteria were defined using the PICO framework. The population included patients with CHD. The intervention was WGS. There was no applicable comparison. The outcome focused on Pathogenic/Likely Pathogenic (P/LP) single nucleotide variants.

Inclusion criteria encompassed patients prenatally or postnatally diagnosed with CHD on imaging, with or without other anomalies or abnormalities (isolated or syndromic CHD), studies with three or more cases of CHD, and variant classification according to the American College of Medical Genetics (ACMG) criteria. Exclusion criteria included studies with fewer than three cases of CHD undergoing WGS, cases not classified according to ACMG criteria, studies on candidate genes, results positive with conventional genetic testing, including karyotype, FISH, and CMA, papers without detailed case information, non‐English papers, reviews, and non‐human studies. Cardiomyopathy cases were excluded. This study focused on single nucleotide variants (SNVs) and did not include copy number variants (CNVs).

### 2.4 Data Extraction and Outcome Measures

Two authors (P.N. and E.S.) independently extracted data using a standardized data collection sheet. Disagreements were resolved through discussion with a third author (H.J.M.). Extracted variables included study characteristics, genome sequencing methodology, variant details, and details on the CHD or any other findings or anomalies.

### 2.5 Quality Assessment

Quality assessment was conducted using modified Standards for Reporting of Diagnostic Accuracy (STARD) criteria, focusing on trio analysis performance, ACMG criteria usage for variant interpretation, and Sanger validation of variants. Quality assessment was performed by two reviewers (E.S. and P.N.), with disagreements resolved by a third party (H.J.M.).

### 2.6 Variant Classification

ACMG classification of genetic variants has two parts: one for the classification of P or LP variants and one for the classification of Benign or Likely Benign variants. Each pathogenic criterion is weighted as very strong (PSV1), strong (PS1‐4), moderate (PM1‐6) or supporting (PP1‐5), and the benign criterion is weighted as stand‐alone (BA1), strong (BS1‐4) or supporting (BP1‐6). The criteria are combined to determine classification from the 5‐tier system: P, LP, variants of uncertain significance (VUS), likely benign, and benign [24].

Genes reported with VUS were classified using the Clinician Genome Resource (ClinGen) as limited, disputed, or definitive [25].

### 2.7 Phenotype Classification

Phenotypes were classified as isolated if CHD was the only anomaly with no other noticeable abnormalities, and as syndromic if there were extracardiac anomalies, developmental abnormalities, or distinctive features.

We also classified based on structural anomalies only (not counting the developmental outcomes) into CHD with or without extracardiac anomalies. The latter was used as a proxy for the classification, possible prenatally, where only structural anomalies and not developmental outcomes can be assessed.

CHD classification included the CHD category and primary diagnosis.

CHD diagnosis was grouped into ten lesion‐based categories, which included:

*Septal defects*: Trial septal defects (ASD), ventricular septal defects (VSD), and atrioventricular septal defects (AVSD)

*Conotruncal defects*: Truncus arteriosus, tetralogy of Fallot (TOF), double outlet right ventricle (DORV), and transposition of great arteries (TGA).

*Left obstructive lesions*: Coarctation of the aorta (CoA), interrupted aortic arch (IAA), bicuspid aortic valve (BAV), Shone's complex, hypoplastic left heart syndrome (HLHS), aortic stenosis (AS), and mitral stenosis (MS).

*Right obstructive lesions*: Pulmonary stenosis (PS), pulmonary atresia (PA), tricuspid atresia (TA), and hypoplastic right heart (HRH).

*Heterotaxy*: As defined by each study.

*Patent ductus arteriosus (PDA)*: Isolated PDA with no other cardiac abnormalities.

*Venous anomaly*: Both systemic and pulmonary venous anomalies are not labeled as heterotaxy.

*Single Ventricle*: Any single ventricle not included in other categories.

*Other*: Including combined lesions (i.e., both AS and PS) and lesions not included elsewhere (isolated dextrocardia, vascular rings, etc.)

*Unspecified*: If CHD diagnosis was not provided.

### 2.8 Statistical Analysis and Data Synthesis

Pooled proportions and 95% confidence intervals of diagnostic yield for single nucleotide variants (SNVs) were calculated for four groups: isolated CHD, CHD with extracardiac structural anomalies, and syndromic CHD. A subgroup analysis was planned to calculate the yield of cases with negative CMA results followed by positive WGS. Heterogeneity was assessed using the Higgins I^2^ test. The inverse variance method was used to minimize imprecision, and a random effects model was applied for pooling effect sizes. Overall significance was tested using a z‐test. Statistical analysis and visualization were performed using RStudio (RStudio Inc., Boston, MA) [26]. Results were summarized in structured tables (Tables 1, 2, 3, 4) and visualized using forest plots (Supporting Information (Figure S1), Supporting Information (Figure S2), Supporting Information (Figure S3), Supporting Information (Figure S4), and Supporting Information (Figure S5)).

**TABLE 1:** Characteristics of the studies included in the systematic review.

Author	Study period	Country	Institute	Study design	Sequencing Method	Inclusion criteria	Exclusion criteria	Diagnostic yield	Total number of cases	CHD number
Hauser (2017) [27]	Not mentioned	USA	Inova translational medicine institute (ITMI)	Prospective	WGS	1 Neonatal and pediatric patients with congenital birth defects of all types, in whom, at the time of enrollment, an underlying etiology for the suspected genetic disorder had not been found using conventional genetic testing methods 2 All infants with cardiac defects without known etiology from that longitudinal cohort study were included in our cardiac subcohort	Fetuses with chromosomal abnormalities or pathogenic/likely pathogenic CNVs detected by conventional tests.	2/34	34	34
Yu (2018) [28]	May 2012–December 2017	China	Beijing Anzhen hospital, capital medical University, Beijing	Retrospective	WGS	Pathology‐confirmed aortopulmonary window (APW) detected through fetal echocardiography. (All cases were associated with other cardiac anomalies)	Not mentioned	0/4	6	6
Alankarage (2019) [29]	Not mentioned	Australia	Victor chang cardiac research institute, sydney; heart center for children, the Children's hospital at westmead, sydney; genetic services of Western Australia, king edward memorial hospital, perth; university of New South Wales, sydney; university of Western Australia, perth; sydney Children's hospital, sydney	Prospective	WGS	Families with probands born with CHD requiring surgical correction; at minimum, a proband–parents trio was sequenced per family. All surgically corrected cases lacking a preexisting genetic diagnosis or otherwise not offered genetic testing were selected for the study without enrichment for a particular mode of inheritance or CHD subtype	Not mentioned	20/97	97	97
Reuter (2020) [30]	2017–2018	Canada	Ted rogers center for heart research, cardiac genome clinic, the hospital for sick children	Prospective	WGS	Families with pediatric heart disease, including laterality defects, outflow tract obstructions, or cardiomyopathies	Known syndromes, metabolic diseases, or medical conditions leading to secondary heart failure	11/111	111	111
Wang (2020) [31]	June 2018–December 2018	China	Children's hospital of fudan university	Retrospective	WGS	Neonate or infant patients in the PICU/NICU and (2) the presence of one of the following items: (1) multisystem failure, (2) congenital cardiac defect, (3) recurrent infection, (4) dysmorphia, (5) metabolic crisis, (6) failure to thrive or early onset developmental delay, and (7) families with an abnormal pregnancy history were included with priority	(1) Parents unavailable or declined to participate and (2) patients who already had an etiologic diagnosis	7/31	130	31
Sweeney (2021) [32]	July 2016 – June 2017	USA	Rady Children's institute for genomic Medicine, San Diego, rady Children's hospital, university of California San Diego	Retrospective	WGS	Inpatient infants at rady Children's hospital without etiologic diagnoses, and in whom a genetic disorder was possible	Fetuses with chromosomal abnormalities or pathogenic/likely pathogenic CNVs detected by conventional tests	10/24	24	24
Munabi (2022) [33]	Not mentioned (retrospective review over a 9‐year period)	USA	Children's Hospital Los Angeles Keck school of medicine of USC operation smile inc. Cedars‐Sinai medical center	Retrospective	WGS	Patients who underwent cleft lip/palate surgery at Children's Hospital Los Angeles Patients with concomitant diagnosis of CHD (outflow tract (OFT)) patients with negative chromosomal microarray analysis No recognizable syndromic association	Patients with a known genetic mutation from diagnostic testing at an outside institution were excluded	3/7	7	7
Cao (2022) [34]	March 2021–December 2021	China	Department of pediatrics, department of obstetrics and gynecology, Chinese university of Hong Kong, laboratory genetics and genomics, fertility preservation research Center, Hong Kong SAR, shenzhen research institute, the Chinese university of Hong Kong, shenzhen, China	Prospective	WGS	Pregnant women with fetal cardiac anomalies detected during routine detailed ultrasound scan for whom conventional testing by karyotyping or CMA were non‐diagnostic	Fetuses with chromosomal abnormalities or pathogenic/likely pathogenic CNVs detected by conventional tests	4/13	13	13
Blue (2022) [35]	Not mentioned	Australia	Heart center for children, sydney medical school, victor chang cardiac research institute, UNSW sydney	Prospective	WGS	Patients with isolated d‐TGA (dextro‐transposition of the great arteries)	Not mentioned	1/100	100	100
Weaver (2022) [36]	Not mentioned	USA	Cincinnati Children's hospital medical center ann & robert H. Lurie Children's hospital of Chicago Icahn school of medicine at mount sinai	Retrospective	WGS and WES	Probands with vPS, pulmonary stenosis, and/or dysplastic pulmonary valve listed as diagnoses. We did not exclude patients with coexisting cardiomyopathy, septal defects or other minor defects	Patients with vPS in conjunction with complex CHD (e.g., conotruncal defects, single ventricle defects, heterotaxy) were excluded.	3/19	119	119
Hays (2023) [37]	June 2020 – March 2022	USA	Columbia university irving medical center	Prospective	WGS	Infants with complex CHD	Individuals with known prenatal genetic diagnoses	9/48	48	48
Yaoita (2024) [38]	Not mentioned	Japan	Tohoku university graduate school of medicine, sendai, Japan; miyagi Children's hospital, sendai, Japan; yamagata university graduate school of medicine, yamagata, Japan	Prospective	WGS	Japanese patients with truncus arteriosus (TA) without 22q11.2 deletion syndrome	22q11.2 deletion syndrome	7/11	11	11
Slavotinek (2024) [39]	Not mentioned	USA	University of California, San Francisco (P3EGS project) HudsonAlpha institute for biotechnology (SouthSeq project) icahn school of medicine at mount sinai and montefiore medical center (NYCKidSeq project)	Prospective	WGS and WES	Pediatric patients with cardiovascular diseases such as arrhythmia, cardiomyopathy, and/or congenital heart disease (CHD)	Not mentioned	76/354	500	354
Austin (2024) [40]	April 2019 – December 2021	Australia	Christopher semsarian, agnes ginges center for molecular cardiology, centenary institute. Murdoch Children's research institute. Royal prince alfred hospital, sydney, NSW, Australia and others	Prospective	WGS	Participants diagnosed with congenital heart disease (CHD), cardiomyopathies, or primary arrhythmias	Deceased individuals or unascertained sudden death. Individuals previously tested with next‐generation sequencing from 2013 onwards. Families with a known pathogenic or likely pathogenic variant	12/80	600	80

Abbreviations: CHD: congenital heart disease, WES: whole exome sequencing, WGS: whole genome sequencing.

**TABLE 2:** Cardiovascular phenotype associations by gene and variant for pathogenic/likely pathogenic variants.

Gene (ClinGen Gene‐Disease Validity Classification when available)	Author and year	CHD classification and main CHD diagnosis	Isolated or syndromic	Variant	NM
CHD7 (13 cases) (definitive, CHARGE syndrome)	Hauser (2017) [27]	Conotruncal lesion, TOF	Syndromic [choanal atresia, hypoplasia of the semicircular canal]	c.5051‐1G>A	NM_017780.3
	Munabi (2022) [33]	Left obstructive lesions, coarctation	Syndromic [bilateral cleft lip palate, left hemifacial microsomia with facial nerve palsy, and an undescended testicle]	c.3378+1G>A	NM_017780.4
	Alankarage (2019) [29]	Conotruncal lesion, DORV	Isolated	c.2098A>G, p.Asn700Asp	NM_017780.3
	Hays (2023) [37]	Conotruncal lesion, TOF	Syndromic [colobomas, left microphthalmia, cleft lip and palate, cryptorchidism, micropenis, intraventricular hemorrhage]	c.1879dup, p.Gln627Profs Ter5	NM_017780.4
	Sweeney (2021) [32]	Left obstructive lesions, BAV/MS	Syndromic [ankyloglossia aspiration, silent aspiration laryngomalacia subglottic stenosis pelviectasis, hydronephrosis]	c.7879C>T, p.Arg2627Ter	N/A
	Slavotinek (2024) [39]	Conotruncal lesion, TOF	Syndromic	c.4185+1G>A	NM_017780.4
	Slavotinek (2024) [39]	Septal defect, AVSD	Syndromic	c.8049del, p.Asp2684 fs	NM_017780.4
	Slavotinek (2024) [39]	Left obstructive lesions, HLHS	Syndromic	c.2919_2922GGAG[1], p.Glu974_Gly975insTer	NM_017780.4
	Slavotinek (2024) [39]	Left obstructive lesions, HLHS	Syndromic	c.7803C>G, p.Tyr2601Ter	NM_017780.4
	Slavotinek (2024) [39]	Septal defect, ASD	Syndromic	c.4393C>T, p.Arg1465Ter	NM_017780.4
	Slavotinek (2024) [39]	Septal defect, VSD	Syndromic	c.3301T>C, p.Cys1101Arg	NM_017780.4
	Austin (2024) [40]	Not specified	Syndromic	c.6883_6884insA, p.(Ser2295Tyrfs*8)	NM_017780.2
	Austin (2024) [40]	Not specified	Syndromic	c.5405‐17G>A	NM_017780.2
PTPN11 (10 cases) (definitive, noonan syndrome)	Reuter (2020) [30]	Other, PS, AI	Isolated	c.209A>G, p.(Lys70Arg)	(NM_002834.4)
	Reuter (2020) [30]	Left obstructive lesions, coarctation	Syndromic [prenatally increased nuchal translucency, short stature, failure to thrive, facial dysmorphisms, ptosis, joint hypermobility/hypotonia, and cryptorchidism.]	c.923A>G, p.(Asn308Ser)	(NM_002834.4)
	Cao (2022) [34]	Right obstructive lesions, PS	Syndromic [polyhydramnios, cerebral ventriculomegaly]	c.1505C>T, p.S502 L	NM_002834.4
	Hays (2023) [37]	Heterotaxy, single ventricle	Syndromic [craniosynostosis, external ear malformation, ventriculomegaly]	c.179G>C, p.Gly60Ala	NM_002834.4
	Weaver (2022) [36]	Right obstructive lesions, PS	Isolated	c.1507G>A, G503 R	N/A
	Weaver (2022) [36]	Right obstructive lesions, PS	Isolated	c.922A>G, N308D	N/A
	Slavotinek (2024) [39]	Other, PS, dysplastic AoV	Syndromic	c.184T>G, p.Tyr62Asp	NM_002834.5
	Slavotinek (2024) [39]	Septal defect, ASD	Syndromic	c.172A>G, p.Asn58Asp	NM_002834.5
	Slavotinek (2024) [39]	Left obstructive lesions, coarctation	Syndromic	c.317A>C, p.Asp106Ala	NM_002834.5
	Austin (2024) [40]	Not specified	Isolated	c.236A>G, p.(Gln79Arg)	NM_002834.3
TMEM260 (7 cases)	Yaoita (2024) [38]	Conotruncal lesion, truncus	Isolated	c.1617del, p.Trp539Cysfs*7	N/A
	Yaoita (2024) [38]	Conotruncal lesion, truncus	Syndromic [posthemorrhagic hydrocephalus, enuresis, myopia, amblyopia, hearing impairment]	c.1617del, p.Trp539Cysfs*7	N/A
	Yaoita (2024) [38]	Conotruncal lesion, truncus	Isolated	c.1617del, p.Trp539Cysfs*7	N/A
	Yaoita (2024) [38]	Conotruncal lesion, truncus	Isolated	c.332dup, p.(Thr112Hisfs*36), c.1617del, p.Trp539Cysfs*7	N/A
	Yaoita (2024) [38]	Conotruncal lesion, truncus	Syndromic [hydronephrosis]	c.1617del, p.Trp539Cysfs*7, c.1960C>T, p.(Gln654*)	N/A
	Slavotinek (2024) [39]	PDA	Isolated	c.377C>T, p.Ala126Val	NM_017799.4
	Slavotinek (2024) [39]	Septal defect, VSD	Syndromic	c.1869+1G>T, c.1869+1G>A (AR)	NM_017799.4
JAG1 (6 cases)	Hauser (2017) [27]	Conotruncal lesion, TOF	Syndromic [jejunal atresia, microcolon preauricular skin tag, cholestasis triangular‐shaped face]	c.551G>A, p.Arg184His	NM_000241.2
	Alankarage (2019) [29]	Septal defect, AVSD	Syndromic [macrocephaly]	c.2429C>T, p.Pro810Leu	NM_000214.2
	Alankarage (2019) [29]	Conotruncal lesion, DORV	Isolated	c.622G>C, p.Gly208Arg	NM_000214.2
	Slavotinek (2024) [39]	Other, PPS	Isolated	c.588C>A, p.Cys196Ter	NM_000214.3
	Austin (2024) [40]	Not specified	Isolated	c.1823_1826del, p.(Gln608Argfs*134)	NM_000214.3
	Austin (2024) [40]	Not specified	Isolated	c.1181dupA, p.(Asn394Lysfs*4)	NM_000214.3
NOTCH1 (6 cases)	Alankarage (2019) [29]	Conotruncal lesion, DORV	Isolated	c.6105del, Ala2036Profs*3	NM_017617.4
	Alankarage (2019) [29]	Right obstructive lesions, hypoplastic right heart	Syndromic [autism, chylous effusion, polyneuropathy, seasonal asthma]	c.5865del, p.Asn1955Lysfs*26	NM_017617.4
	Alankarage (2019) [29]	Conotruncal lesion, DORV	Syndromic [right foot: 2nd 3rd toes, absent middle and distal phalanges; 4th toe, hypoplastic middle phalanges, absent distal phalanges; 5th toe, hypoplastic middle and distal phalanges. Left foot: 2nd and 3rd toes, hypoplastic middle]	c.4416C>G, p.Cys1472Trp	NM_017617.4
	Yaoita (2024) [38]	Conotruncal lesion, truncus	Isolated	c.545G>A, p.(Cys182Tyr)	N/A
	Weaver (2022) [36]	Right obstructive lesions, PS	Isolated	c.4918del, A1640Hfs*9	N/A
	Austin (2024) [40]	Not specified	Isolated	c.5496_5505delinsGGT, p.(Asp1833Valfs*52)	NM_017617.5
KMT2D (5 cases) (definitive, kabuki syndrome)	Reuter (2020) [30]	Left obstructive lesions, shones	Syndromic [developmental delay (gross motor, speech and language), failure to thrive, tracheal tug on inspiration, high palate, triangular face, ptosis, hyperteloric, protruding forward facing ears, sacral dimple, eczema, and dysmorphisms]	c.15673C>T, p.(Arg5225Cys)	(NM_003482.3)
	Hays (2023) [37]	Left obstructive lesions, HLHS	Syndromic [microcephaly, dysmorphic features]	c.9880_9894delinsCCCTGCC, p.Ala3294Prof s*4	NM_003482.3
	Sweeney (2021) [32]	Right obstructive lesions, PS	Syndromic [short neck redundant skin in nuchal area mild retrognathia thrombocytopenia hyperthyroidism neonatal graves' disease]	c.3228_3230delGAA, p.Lys1077del	N/A
	Slavotinek (2024) [39]	Septal defect, VSD	Syndromic	c.7933C>T, p.Arg2645Ter	NM_003482.3
	Slavotinek (2024) [39]	Left obstructive lesions, coarctation	Syndromic	c.15079C>T, p.Arg5027Ter	NM_003482.3
NODAL (4 cases)	Alankarage (2019) [29]	Single ventricle, single ventricle	Isolated	c.123_142dup, Tyr48Trpfs*5	NM_018055.4
	Alankarage (2019) [29]	Heterotaxy, single ventricle	Syndromic [asplenia]	c.123_142dup, p.Tyr48Trpfs*5	NM_018055.4
	Alankarage (2019) [29]	Conotruncal lesion, TGA	Isolated	c.919C>T, p.Arg307	NM_018055.4
	Austin (2024) [40]	Not specified	Isolated	c.158_165del, p.(Pro53Argfs*23)	NM_018055.5
DNAH5 (3 cases) (definitive, primary ciliary dyskinesia)	Cao (2022) [34]	Other, dextrocardia	Syndromic [situs inversus]	c.5563dupA, p.I1855 fs, c.6442C>T, p.Q2148X	NM_001369.2
	Slavotinek (2024) [39]	Heterotaxy, dextrocardia	Syndromic	c.10815del, p.Pro3606 fs, c.11028+5G>A	NM_001369.3, NM_001369.2
	Slavotinek (2024) [39]	Other, dextrocardia	Syndromic	c.13486C>T, p.Arg4496Ter	NM_001369.2
GATA6 (3 cases) (definitive, CHD)	Alankarage (2019) [29]	Single ventricle, single ventricle	Isolated	c.1595_1596del, p.Pro532Hisfs*100	NM_005257.5
	Yaoita (2024) [38]	Conotruncal lesion, truncus	Syndromic [shortening of the lingual pedicel, left migrating testis, left polydactyly]	c.1367G>A, p.Arg456His	N/A
	Slavotinek (2024) [39]	Septal defect, VSD	Syndromic	c.1367G>A, p.Arg456His	NM_005257.5
NF1 (3 cases) (definitive, neurofibromatosis type 1)	Alankarage (2019) [29]	Conotruncal lesion, TOF	Isolated	c.3560T>G, p.Leu1187Arg	NM_000267.3
	Sweeney (2021) [32]	Right obstructive lesions, PA/IVS	Isolated	c.5118delT, p.Val1707PhefsTer3	N/A
	Slavotinek (2024) [39]	PDA	Syndromic	c.1845+1G>A	NM_000267.3
ARID1B (2 cases) (definitive, coffin‐siris syndrome)	Sweeney (2021) [32]	Left obstructive lesions, Shone's	Syndromic [small for gestational age congenital diaphragmatic hernia congenital heart disease hypoplasia of corpus callosum abnormal face shape recurrent respiratory infections delayed gross motor development]	c.3096_3100delCAAAG, p.Lys1033ArgfsTer32	N/A
	Slavotinek (2024) [39]	PDA	Syndromic	c.1293_1317del, p.Ala433 fs	NM_001374828.1
MYH6 (2 cases) (definitive, CHD)	Alankarage (2019) [29]	Left obstructive lesions, coarctation	Isolated	c.731G>A, p.Arg244His, c.5794A>T, Lys1932*	NM_002471.3
	Hays (2023) [37]	Conotruncal lesion, DORV	Syndromic [pentalogy of cantrell]	c.1138G>A, p.Glu380Lys	NM_002471.3
COL2A1 (2 cases) (definitive stickler syndrome)	Slavotinek (2024) [39]	Septal defect, ASD	Syndromic	c.1826G>T, p.Gly609Val	NM_001844.5
	Slavotinek (2024) [39]	PDA	Syndromic	c.2594del, p.Pro865 fs	NM_001844.5
FOXF1 (2 cases)	Hays (2023) [37]	Left obstructive lesions, coarctation	Syndromic [omphalocele, respiratory failure]	c.691_698del, p.Ala231Argfs Ter61	NM_001451.3
	Sweeney (2021) [32]	Venous abnormality, PAPVR	Isolated	c.188G>T, p.Ser63Ile	N/A
GATA4 (2 cases) (definitive, CHD)	Alankarage (2019) [29]	Septal defect, VSD	Syndromic [hyperkalaemia, diffuse white matter disease, mild global developmental delay at 2 years, focal weakness of left leg]	c.959G>A, p.Arg320Gln	NM_002052.4
	Austin (2024) [40]	Not specified	Isolated	c.851_853delGCA, p.(Arg284_Asn285delinsHis)	NM_002052.4
GREB1L (2 cases)	Slavotinek (2024) [39]	Septal defect, VSD	Syndromic	c.3977del, p.Lys1326 fs, c.3983del, p.Gly1328 fs	NM_001142966.3
	Slavotinek (2024) [39]	Conotruncal lesion, TOF	Syndromic	c.157+1delG	NM_001142966.2
MYBPC3 (2 cases) (definitive, hypertrophic cardiomyopathy)	Sweeney (2021) [32]	Right obstructive lesions, PA/IVS	Isolated	c.3184delG, p.Val1062LeufsTer13	N/A
	Blue (2022) [35]	Conotruncal lesion, TGA	Isolated	c.2526C>G, p.Tyr842Ter	NM_000256.3
MYRF (2 cases)	Slavotinek (2024) [39]	Other, dextrocardia	Syndromic	c.1200C>A, p.Asn400Lys	NM_001127392.3
	Slavotinek (2024) [39]	Left obstructive lesions, HLHS	Isolated	c.2074C>G, p.Leu692Val	NM_001127392.3
ZEB2 (2 cases) (definitive, mowat‐wilson syndrome)	Sweeney (2021) [32]	Right obstructive lesions, PS	Syndromic [aganglionic megacolon generalized hypotonia coronal hypospadias abnormality of corpus callosum abnormality of philtrum]	c.656delG, p.Gly219AlafsTer5	N/A
	Slavotinek (2024) [39]	Left obstructive lesions, BAV	Syndromic	c.2672_2678delCCAAACC	NM_014795.3
FLT4 (2 cases) (definitive, CHD)	Austin (2024) [40]	Not specified	Isolated	c.1755C>G, p.(Tyr585*)	NM_182925.4
	Reuter (2020) [30]	Conotruncal lesion, TOF	Syndromic [mild congenital lymphedema]	c.89delC, p.(Pro30Argfs*3)	(NM_182925.4)
ACADVL (1 case) (definitive, very long chain Acyl‐CoA dehydrogenase deficiency)	Slavotinek (2024) [39]	Left obstructive lesions, coarctation	Isolated	c.507_527del, p.Met169_Gly175del	NM_000018.4
ACTC1 (1 case) (definitive, hypertrophic cardiomyopathy)	Alankarage (2019) [29]	Septal defect, ASD	Syndromic [cleft palate]	c.203C>T, p.Thr68Ile	NM_005159.4
ACVR2B (1 case)	Alankarage (2019) [29]	Conotruncal lesion, TOF	Syndromic [esophageal atresia, duodenal atresia, tracheoesophageal fistula, imperforate anus with mucous fistula, right multi‐cystic dysplastic kidney with bilateral hydronephrosis, severe penoscrotal hypospadias, vertebral defects, bilateral fixed talipes deformities]	c.1057G>T, p.Gly353Trp	NM_001106.3
ANK1 (1 case) (definitive, hereditary spherocytosis)	Slavotinek (2024) [39]	Septal defect, VSD	Syndromic	c.4000C>T, p.Arg1334Ter	NM_000037.4
ANKRD11 (1 case) (definitive, KBG syndrome)	Slavotinek (2024) [39]	Left obstructive lesions, HLHS	Syndromic	c.2408_2412delAAAAA, Lys803 fs	NM_013275.5
ANKRD11 (1 case) (definitive, KBG syndrome)	Reuter (2020) [30]	Septal defect, AVSD	Syndromic [feeding difficulties, polyhydramnion, dilated bowel, renal cyst, choroid plexus cyst, micrognathia]	c.5238_5239delGC, p.(Pro1747Argfs*49)	(NM_013275.5)
ARID1A (1 case) (definitive, coffin‐siris syndrome)	Slavotinek (2024) [39]	Single ventricle, mitral atresia, DORV, coarctation	Syndromic	c.750_771dup, p.Ser258 fs	NM_006015.6
ARID2 (1 case) (definitive, coffin‐siris syndrome)	Slavotinek (2024) [39]	Septal defect, VSD	Syndromic	c.109dup, p.Ile37 fs	NM_152641.4
BCOR (1 case) (definitive, microphthalmia syndrome)	Alankarage (2019) [29]	Septal defect, ASD	Syndromic [congenital cataract glaucoma, global developmental delay, amblyopia]	c. 2488_2489del, p.Ser830Cysfs*6	NM_001123383.1
BMP2 (1 case)	Slavotinek (2024) [39]	PDA	Syndromic	c.939G>A, p.Trp313Ter	NM_001200.4
BRPF1 (1 case)	Slavotinek (2024) [39]	Other, right arch	Syndromic	c.1755_1756delinsT, p.Lys585 fs	NM_001003694.2
CDK13 (1 case) (definitive, syndromic intellectual disability)	Austin (2024) [40]	Not specified	Isolated	c.484dupG, p.(Ala357Serfs*11)	NM_003718.4
CEP120 (1 case)	Slavotinek (2024) [39]	Septal defect, AVSD	Syndromic	c.2323C>T, p.Gln775Ter	NM_153223.3
CFC1 (1 case)	Alankarage (2019) [29]	Left obstructive lesions, HLHS	Isolated	c.522del, p.Ala175Argfs*56	NM_032545.3
COL1A2 (1 case) (definitive osteogenesis imperfect and ehlers‐danlos syndrome arthrochalasia type)	Slavotinek (2024) [39]	Septal defect, ASD	Syndromic	c.1072G>A, p.Gly358Ser	NM_000089.3
COL4A1 (1 case)	Cao (2022) [34]	Right obstructive lesions, PS	Syndromic [suspected fetal IVH, bilateral lateral ventricle heterogeneity]	c.2281G>A, p.G761 R	NM_001845.6
CREBBP (1 case) (definitive, rubinstein‐taybi syndrome)	Slavotinek (2024) [39]	Septal defect, VSD	Syndromic	c.85+1G>A	NM_004380.3
CYP11A1 (1 case)	Slavotinek (2024) [39]	Septal defect, ASD	Syndromic	c.508_509del, p.Leu170 fs	NM_000781.3
DLL4 (1 case)	Slavotinek (2024) [39]	Conotruncal lesion, truncus	Isolated	c.1857_1864del, p.Pro621 fs	NM_019074.4
DOCK6 (1 case) (definitive, adams‐oliver syndrome)	Alankarage (2019) [29]	Conotruncal lesion, TOF	Syndromic [esophageal atresia, duodenal atresia, tracheoesophageal fistula, imperforate anus with mucous fistula, right multi‐cystic dysplastic kidney with bilateral hydronephrosis, severe penoscrotal hypospadias, vertebral defects, bilateral fixed talipes deformities]	c.3163G>A, Val1055Met	NM_020812.3
EFTUD2 (1 case) (definitive, madibulofacial dysostosis‐mircocephaly syndrome)	Slavotinek (2024) [39]	Septal defect, VSD	Syndromic	c.2335C>T, p.Leu779Phe	NM_004247.4
EHMT1 (1 case) (definitive, kleefstra syndrome)	Slavotinek (2024) [39]	Septal defect, ASD	Syndromic	c.3310G>A, p.Glu1104Lys	NM_024757.4
ELN (1 case) (definitive, cutis laxa)	Austin (2024) [40]	Not specified	Isolated	c.1069_1091del, p.(Ala357Serfs*11)	NM_000501.3
ERF (1 case) (definitive, craniosynostosis 4)	Slavotinek (2024) [39]	PDA	Syndromic	c.517_521dup, p.Ala175 fs	NM_006494.4
FAM111 A (1 case)	Slavotinek (2024) [39]	PDA	Syndromic	c.931A>T, p.Ile311Phe	NM_001312909.2
FOXJ1 (1 case) (definitive, primary ciliary dyskinesia)	Hays (2023) [37]	Heterotaxy, TAPVR	Syndromic [fetal growth restriction, heterotaxy]	c.867dup, p.Ser290Glnfs Ter12	NM_001454.4
GRIN2B (1 case) (definitive, complex neurodevelopmental disorder)	Slavotinek (2024) [39]	PDA	Syndromic	c.2045G>A, p.Arg682His	NM_000834.3
HDAC8 (1 case) (definitive, cornelia de lange syndrome)	Slavotinek (2024) [39]	Septal defect, AVSD	Syndromic	c.490C>T, p.Arg164Ter	NM_018486.2
HNRNPK (1 case) (definitive neurodevelopmental disorder‐ craniofacial dysmorphism‐cardiac defect‐hip dysplasia syndrome)	Slavotinek (2024) [39]	Septal defect, VSD	Syndromic	c.646‐1G>A	NM_031263.4
INVS (1 case) (definitive, nephronophthisis 2)	Alankarage (2019) [29]	Heterotaxy, unbalanced AVSD	Isolated	c.3182dup, Asn1061Lysfs*20	NM_014425.4
KRAS (1 case) (definitive, noonan syndrome; strong, cardiofaciocutaneous syndrome	Cao (2022) [34]	Venous abnormality, venous abnormality	Syndromic [dilated bilateral renal pelvis, and overgrowth]	c.178G>A, p.G60S	NM_004985.5
MADD (1 case)	Slavotinek (2024) [39]	PDA	Syndromic	c.3070C>T, p.Gln1024Ter	NM_003682.4
MAGEL2 (1 case) (definitive, schaaf‐yang syndrome)	Slavotinek (2024) [39]	Septal defect, ASD	Syndromic	c.1912C>T, p.Gln638Ter	NM_019066.5
MED12 (1 case) (definitive, MED12‐related intellectual disability syndrome)	Munabi (2022) [33]	Left obstructive lesions, coarctation	Syndromic [bilateral preauricular pits, hearing loss, ectopic ureters, hydronephrosis, intestinal malrotation with ladd bands]	c.3551del, p.Gln1184ArgfsTer14	NM_005120.3
MED13 (1 case) (definitive, complex neuordevelopmental disorder)	Slavotinek (2024) [39]	Left obstructive lesions, AS	Syndromic	c.4198C>T, p.Arg1400Ter	NM_005121.2
MMUT (1 case) (definitive, methylmalonic aciduria due to Methylmalonyl‐CoA mutase deficiency)	Slavotinek (2024) [39]	Septal defect, ASD	Syndromic	c.91C>T, p.Arg31Ter	NM_000255.4
MT‐ATP6 (1 case) (definitive, leigh syndrome)	Hays (2023) [37]	Left obstructive lesions, coarctation	Syndromic [PAH, bilateral hydronephrosis, epispadias, cryptorchidism, micropenis, dysplastic nails, thrombocytopenia, choroid plexus cyst]	m.8969G>A, p.Ser148Asn	NC_012920.1
MT‐TL1 (1 case) (definitive, mitochondrial disease)	Slavotinek (2024) [39]	Left obstructive lesions, HLHS	Syndromic	m:3243A>G	NC_012920.1
MYH11 (1 case) (definitive, familial thoracic aortic aneurysm and aortic dissection)	Reuter (2020) [30]	PDA	Isolated	c.4578+1G>A, p.?	(NM_002474.2)
MYOD1 (1 case)	Slavotinek (2024) [39]	Other, dysplastic TV	Syndromic	c.188C>A, p.Ser63Ter	NM_002478.5
NADYSN1 (1 case)	Slavotinek (2024) [39]	Other, PPS	Syndromic	c.1459C>T, p.Arg487Ter	NM_018161.5
NEXMIF (1 case) (definitive, X‐linked complex neurodevelopmental disorder)	Reuter (2020) [30]	Left obstructive lesions, AS	Syndromic [global developmental delay, self‐injurious behavior, microcephaly, short stature, hypotonia, joint laxity, and dysmorphic features]	c.1502delG, p.(Gly501Valfs*4)	(NM_001008537.2)
NIPBL (1 case) (definitive, cornelia de lange syndrome)	Reuter (2020) [30]	Single ventricle, unbalanced AVSD	Syndromic [borderline microcephaly, global developmental delay, hypotonia]	c.771+1G>A, p.?	(NM_133433.3)
NKX2 (1 case)	Austin (2024) [40]	Not specified	Isolated	c.487delC, p.(Leu163Cysfs*13)	NM_004387.3
NKX2‐5 (1 case) (definitive, NKX2.5‐related congenital, conduction and myopathic heart disease)	Slavotinek (2024) [39]	Conotruncal lesion, TOF	Isolated	c.439del, p.Gln147 fs	NM_004387.4
NOTCH2 (1 case)	Slavotinek (2024) [39]	Septal defect, ASD	Syndromic	c.6909del, p.Ile2304 fs	NM_024408.4
NR2F2 (1 case) (definitive, CHD)	Reuter (2020) [30]	Left obstructive lesions, coarctation	Syndromic [macrocephaly, hemangioma, scoliosis, intellectual disability.]	c.671T>A, p.(Val224Asp)	(NM_021005.3)
PACS1 (1 case) (definitive, schuurs‐hoeijmakers syndrome)	Slavotinek (2024) [39]	Septal defect, VSD	Syndromic	c.607C>T, p.Arg203Trp	NM_018026.3
PEX26 (1 case) (definitive, peroxisome biogenesis disorder)	Slavotinek (2024) [39]	PDA	Syndromic	c.34dup, p.Leu12 fs	NM_001127649.3
PHEX (1 case)	Sweeney (2021) [32]	Left obstructive lesions, coarctation	Syndromic [ambiguous genitalia, male penoscrotal hypospadias chordee sacral dimple abnormality of the vertebrae knee flexion contractures in utero growth restriction mild micrognathia thrombocytopenia congenital thrombocytopenia]	c.1604C>T, p.Thr535Met	N/A
PHF6 (1 case) (definitive, borjeson‐forssman‐lehmann syndrome)	Slavotinek (2024) [39]	Septal defect, ASD	Syndromic	c.1024C>T, p.Arg342Ter	NM_032458.2
PHOX2B (1 case) (definitive, haddad syndrome)	Slavotinek (2024) [39]	Septal defect, ASD	Syndromic	c.765_779dup, p.Ala256_Ala260dup	NM_003924.3
PIGN (1 case) (definitive, multiple congenital anomalies‐hypotonia seizures syndrome 1)	Slavotinek (2024) [39]	Septal defect, VSD	Syndromic	c.1557G>A, p.Trp519Ter	NM_176787.5
PKD1 (1 case) (definitive, polycystic kidney disease)	Hays (2023) [37]	Left obstructive lesions, coarctation	Syndromic [dysmorphic features]	c.6795C>G, p.Tyr2265Ter	NM_001009944.2
POGZ (1 case) (definitive, intellectual disability‐microcephaly‐strabismus‐behavioral abnormalities syndrome)	Reuter (2020) [30]	Left obstructive lesions, HLHS	Syndromic [global developmental delay, hypotonia, borderline short stature and gastroesophageal reflux]	c.3403delG, p.(Glu1135Argfs*3)	(NM_015100.3)
POLR1C (1 case) (moderate, treacher collins syndrome)	Sweeney (2021) [32]	Left obstructive lesions, BAV	Syndromic [prolonged QT syndrome prolonged QTc syndrome pulmonary hypertension hip dysplasia congenital microcephaly feeding difficulties aspiration failure to thrive]	c.242T>C, p.Leu81Pro, c.326G>A, p.Arg109His	N/A
PRR12 (1 case) (definitive, neuroocular syndrome)	Slavotinek (2024) [39]	Left obstructive lesions, coarctation	Syndromic	c.2680_2695dup, p.Val899AlafsTer43	N/A
PUF60 (1 case) (definitive, syndromic intellectual disability)	Hays (2023) [37]	Conotruncal lesion, DORV	Syndromic [refractive error, renal duplication]	c.24+1G>A	NM_078480.2
PURA (1 case) (definitive, complex neurodevelopmental disorder)	Reuter (2020) [30]	Septal defect, VSD	Syndromic [developmental delay, infantile spasms, hypoventilation, macrosomia, macrocephaly, hypotonia]	c.812_814delTCT, p.(Phe271del)	(NM_005859.4)
RFXANK (1 case) (definitive, MHC class II deficiency)	Slavotinek (2024) [39]	Right obstructive lesions, tricuspid atresia	Syndromic	c.454_455del, p.Ile152 fs	NM_003721.4
RRAS2 (1 case) (definitive, noonan syndrome)	Slavotinek (2024) [39]	Septal defect, VSD	Syndromic	c.70_78dup, p.Gly24_Gly26dup	NM_012250.6
SCN1A (1 case) (definitive, dravet syndrome)	Slavotinek (2024) [39]	PDA	Syndromic	c.707T>C, p.Ile236Thr	NM_006920.5
SCN4A (1 case) (definitive, SCN4A‐related myopathy)	Slavotinek (2024) [39]	Left obstructive lesions, IAA	Syndromic	c.4364T>C, p.Ile1455Thr	NM_000334.4
SETBP1 (1 case) (definitive, schinzel‐giedion syndrome and complex neurodevelopmental disorder)	Slavotinek (2024) [39]	PDA	Syndromic	c.2608G>A, p.Gly870Ser	NM_015559.3
SHOC2 (1 case) (definitive, noonan syndrome)	Slavotinek (2024) [39]	Right obstructive lesions, PS	Syndromic	c.4A>G, p.Ser2Gly	NM_007373.3
SLC25A1 (1 case) (definitive, mitochondrial disease)	Slavotinek (2024) [39]	Septal defect, VSD	Syndromic	c.844C>T, p.Arg282Cys, c.821+1G>A	NM_005984.5
SMAD6 (1 case)	Alankarage (2019) [29]	Left obstructive lesions, HLHS	Isolated	c.86del, p.Gly29Alafs*35	NM_005585.4
SMARCA4 (1 case) (definitive, coffin‐siris syndrome)	Munabi (2022) [33]	Left obstructive lesions, coarctation	Syndromic [isolated cleft palate, multiple dental caries, diminished hearing, and a left eye larger than right. Other anomalies included a left inguinal hernia and right hydronephrosis]	c.2936G>A, p.Arg979Gln	NM_003072.5
SMC1A (1 case) (definitive, X‐linked complex neurodevelopmental disorder)	Slavotinek (2024) [39]	PDA	Syndromic	c.3592G>A, p.Glu1198Lys	NM_006306.4
SNRPB (1 case)	Slavotinek (2024) [39]	Septal defect, VSD	Syndromic	c.155 + 301G>C	NM_003091.4
TFAP2A (1 case)	Slavotinek (2024) [39]	PDA	Syndromic	c.752A>G, p.Arg251Gly	NM_001042425.2
TGFBI (1 case)	Slavotinek (2024) [39]	Right obstructive lesions, PS	Syndromic	c.1663C>T, p.Arg555Trp	NM_000358.3
TK2 (1 case) (definitive, mitochondrial disease)	Slavotinek (2024) [39]	Septal defect, ASD	Syndromic	c.129_132del, p.Lys43 fs	NM_004614.5
TLL1 (1 case) (limited, CHD)	Alankarage (2019) [29]	Left obstructive lesions, Shone's	Isolated	c.2578 A>G, p.Thr860Ala	NM_012464.4
TMEM70 (1 case) (definitive, mitochondrial disease)	Slavotinek (2024) [39]	Left obstructive lesions, coarctation	Isolated	c.317–2A>G	NM_017866.6
TP63 (1 case)	Slavotinek (2024) [39]	Left obstructive lesions, coarctation	Syndromic	c.739C>T, p.His247Tyr	NM_003722.5
TPM1 (1 case) (definitive, hypertrophic cardiomyopathy)	Sweeney (2021) [32]	Single ventricle, other hypoplastic LV	Syndromic [hydrops fetalis fetal ascites pericardial effusion]	c.533G>A, p.Arg178His	N/A
TSC2 (1 case) (definitive, tuberous sclerosis)	Sweeney (2021) [32]	Other, rhabdomyoma	Syndromic [hamartoma of the eye cerebral hamartomas]	c.935_936delTC, p.Leu312GlnfsTer25	N/A
TTPA (1 case)	Slavotinek (2024) [39]	Left obstructive lesions, BAV	Syndromic	c.552G>A, p.Thr184 =	NM_000370.3
ZC4H2 (1 case) (definitive, X‐linked syndromic intellectual disability)	Slavotinek (2024) [39]	Septal defect, VSD	Syndromic	c.412C>T, p.Gln138Ter	NM_018684.4
ZIC3 (1 case)	Slavotinek (2024) [39]	Conotruncal lesion, TOF	Syndromic	c.1103del, p.Arg368ProfsTer40	NM_003413.4

Abbreviations: AI, aortic insufficiency; AoV, aortic valve; ASD, atrial septal defect; AVSD, atrioventricular septal defect; BAV, bicuspid aortic valve; CHD, congenital heart disease; DORV, double outlet right ventricle; HLHS, hypoplastic left heart syndrome; IAA, interrupted aortic arch; IVH, Intraventricular Hemorrhage; LV, left ventricle; MS, mitral stenosis; PA/IVS, Pulmonary Atresia with Intact Ventricular Septum; PAPVR, partial anomalous pulmonary venous return; PDA, patent ductus arteriosus; PPS, peripheral pulmonary stenosis; PS, pulmonary stenosis; TAPVR, total anomalous pulmonary venous return; TGA, transposition of the great arteries; TOF, tetralogy of Fallot; TV, tricuspid valve; VSD, ventricular septal defect.

**TABLE 3:** Cardiovascular phenotype associations by gene and variant for pathogenic/likely pathogenic variants.

CHD classification	Main CHD diagnosis	Author and year	Isolated or syndromic	Gene	Variant	NM
Septal defect [29, 30, 39] (*n* = 37)	VSD (*n* = 18)	Slavotinek (2024) [39]	Syndromic	ANK1	c.4000C>T, p.Arg1334Ter	NM_000037.4
		Slavotinek (2024) [39]	Syndromic	ARID2	c.109dup, p.Ile37 fs	NM_152641.4
		Slavotinek (2024) [39]	Syndromic	CHD7	c.3301T>C, p.Cys1101Arg	NM_017780.4
		Slavotinek (2024) [39]	Syndromic	CREBBP	c.85+1G>A	NM_004380.3
		Slavotinek (2024) [39]	Syndromic	EFTUD2	c.2335C>T, p.Leu779Phe	NM_004247.4
		Alankarage (2019) [29]	Syndromic [hyperkalaemia, diffuse white matter disease, mild global developmental delay at 2 years, focal weakness of left leg]	GATA4	c.959G>A, p.Arg320Gln	NM_002052.4
		Slavotinek (2024) [39]	Syndromic	GATA6	c.1367G>A, p.Arg456His	NM_005257.5
		Slavotinek (2024) [39]	Syndromic	GREB1L, GREB1L	c.3977del, p.Lys1326 fs, c.3983del, p.Gly1328 fs	NM_001142966.3, NM_001142966.3
		Slavotinek (2024) [39]	Syndromic	HNRNPK	c.646‐1G>A	NM_031263.4
		Slavotinek (2024) [39]	Syndromic	KMT2D	c.7933C>T, p.Arg2645Ter	NM_003482.3
		Slavotinek (2024) [39]	Syndromic	PACS1	c.607C>T, p.Arg203Trp	NM_018026.3
		Slavotinek (2024) [39]	Syndromic	PIGN	c.1557G>A, p.Trp519Ter	NM_176787.5
		Reuter (2020) [30]	Syndromic [developmental delay, infantile spasms, hypoventilation, macrosomia, macrocephaly, hypotonia]	PURA	c.812_814delTCT, p.(Phe271del)	NM_005859.4
		Slavotinek (2024) [39]	Syndromic	RRAS2	c.70_78dup, p.Gly24_Gly26dup	NM_012250.6
		Slavotinek (2024) [39]	Syndromic	SLC25A1	c.844C>T, p.Arg282Cys, c.821+1G>A	NM_005984.5, NM_005984.5
		Slavotinek (2024) [39]	Syndromic	SNRPB	c.155 + 301G>C	NM_003091.4
		Slavotinek (2024) [39]	Syndromic	TMEM260, TMEM260	c.1869+1G>T, c.1869+1G>T	NM_017799.4, NM_017799.4
		Slavotinek (2024) [39]	Syndromic	ZC4H2	c.412C>T, p.Gln138Ter	NM_018684.4
	ASD (*n* = 14)	Alankarage (2019) [29]	Syndromic [congenital cataract glaucoma, global developmental delay, amblyopia]	ACTC1	c.203C>T, p.Thr68Ile	NM_005159.4
		Alankarage (2019) [29]	Syndromic [cleft palate]	BCOR	c. 2488_2489del, p.Ser830Cysfs*6	NM_001123383.1
		Slavotinek (2024) [39]	Syndromic	CHD7	c.4393C>T, p.Arg1465Ter	NM_017780.4
		Slavotinek (2024) [39]	Syndromic	COL1A2	c.1072G>A, p.Gly358Ser	NM_000089.3
		Slavotinek (2024) [39]	Syndromic	COL2A1	c.1826G>T, p.Gly609Val	NM_001844.5
		Slavotinek (2024) [39]	Syndromic	CYP11A1	c.508_509del, p.Leu170 fs	NM_000781.3
		Slavotinek (2024) [39]	Syndromic	EHMT1	c.3310G>A, p.Glu1104Lys	NM_024757.4
		Slavotinek (2024) [39]	Syndromic	MAGEL2	c.1912C>T, p.Gln638Ter	NM_019066.5
		Slavotinek (2024) [39]	Syndromic	MMUT	c.91C>T, p.Arg31Ter	NM_000255.4
		Slavotinek (2024) [39]	Syndromic	NOTCH2	c.6909del, p.Ile2304 fs	NM_024408.4
		Slavotinek (2024) [39]	Syndromic	PHF6	c.1024C>T, p.Arg342Ter	NM_032458.2
		Slavotinek (2024) [39]	Syndromic	PHOX2B	c.765_779dup, p.Ala256_Ala260dup	NM_003924.3
		Slavotinek (2024) [39]	Syndromic	PTPN11	c.172A>G, p.Asn58Asp	NM_002834.5
		Slavotinek (2024) [39]	Syndromic	TK2	c.129_132del, p.Lys43 fs	NM_004614.5
	AVSD (*n* = 5)	Reuter (2020) [30]	Syndromic [feeding difficulties, polyhydramnion, dilated bowel, renal cyst, choroid plexus cyst, micrognathia]	ANKRD11	c.5238_5239delGC, p.(Pro1747Argfs*49)	(NM_013275.5)
		Slavotinek (2024) [39]	Syndromic	CEP120	c.2323C>T, p.Gln775Ter	NM_153223.3
		Slavotinek (2024) [39]	Syndromic	CHD7	c.8049del, p.Asp2684 fs	NM_017780.4
		Slavotinek (2024) [39]	Syndromic	HDAC8	c.490C>T, p.Arg164Ter	NM_018486.2
		Alankarage (2019) [29]	Syndromic [macrocephaly]	JAG1	c.2429C>T, p.Pro810Leu	NM_000214.2
Left obstructive lesions [29, 30, 32, 33, 37, 39](*n* = 35)	Coarctation (*n* = 16)	Slavotinek (2024) [39]	Isolated	ACADVL	c.507_527del, p.Met169_Gly175del	NM_000018.4
		Munabi (2022) [33]	Syndromic [bilateral cleft lip palate, left hemifacial microsomia with facial nerve palsy, and an undescended testicle]	CHD7	c.3378+1G>A	NM_017780.4
		Hays (2023) [37]	Syndromic [omphalocele, respiratory failure]	FOXF1	c.691_698del, p.Ala231Argfs Ter61	NM_001451.3
		Slavotinek (2024) [39]	Syndromic	KMT2D	c.15079C>T, p.Arg5027Ter	NM_003482.3
		Munabi (2022) [33]	Syndromic [bilateral preauricular pits, hearing loss, ectopic ureters, hydronephrosis, intestinal malrotation with ladd bands]	MED12	c.3551del, p.Gln1184ArgfsTer14	NM_005120.3
		Hays (2023) [37]	Syndromic [PAH, bilateral hydronephrosis, epispadias, cryptorchidism, micropenis, dysplastic nails, thrombocytopenia, choroid plexus cyst]	MT‐ATP6	m.8969G>A, p.Ser148Asn	NC_012920.1
		Alankarage (2019) [29]	Isolated	MYH6, MYH6	c.731G>A, p.Arg244His, c.5794A>T, Lys1932*	NM_002471.3
		Reuter (2020) [30]	Syndromic [macrocephaly, hemangioma, scoliosis, intellectual disability.]	NR2F2	c.671T>A, p.(Val224Asp)	(NM_021005.3)
		Sweeney (2021) [32]	Syndromic [ambiguous genitalia, male penoscrotal hypospadias chordee sacral dimple abnormality of the vertebrae knee flexion contractures in utero growth restriction mild micrognathia thrombocytopenia congenital thrombocytopenia]	PHEX	c.1604C>T, p.Thr535Met	N/A
		Hays (2023) [37]	Syndromic [dysmorphic features]	PKD1	c.6795C>G, p.Tyr2265Ter	NM_001009944.2
		Slavotinek (2024) [39]	Syndromic	PRR12	c.2680_2695dup, p.Val899AlafsTer43	
		Slavotinek (2024) [39]	Syndromic	PTPN11	c.317A>C, p.Asp106Ala	NM_002834.5
		Reuter (2020) [30]	Syndromic [prenatally increased nuchal translucency, short stature, failure to thrive, facial dysmorphisms, ptosis, joint hypermobility/hypotonia, and cryptorchidism]	PTPN11	c.923A>G, p.(Asn308Ser)	(NM_002834.4)
		Munabi (2022) [33]	Syndromic [isolated cleft palate, multiple dental caries, diminished hearing, and a left eye larger than right. Other anomalies included a left inguinal hernia and right hydronephrosis]	SMARCA4	c.2936G>A, p.Arg979Gln	NM_003072.5
		Slavotinek (2024) [39]	Isolated	TMEM70	c.317–2A>G	NM_017866.6
		Slavotinek (2024) [39]	Syndromic	TP63	c.739C>T, p.His247Tyr	NM_003722.5
	HLHS (*n* = 9)	Slavotinek (2024) [39]	Syndromic	ANKRD11	c.2408_2412delAAAAA, Lys803 fs	NM_013275.5
		Alankarage (2019) [29]	Isolated	CFC1	c.522del, p.Ala175Argfs*56	NM_032545.3
		Slavotinek (2024) [39]	Syndromic	CHD7	c.2919_2922GGAG[1], p.Glu974_Gly975insTer	NM_017780.4
		Slavotinek (2024) [39]	Syndromic	CHD7	c.7803C>G, p.Tyr2601Ter	NM_017780.4
		Hays (2023) [37]	Syndromic [microcephaly, dysmorphic features]	KMT2D	c.9880_9894delinsCCCTGCC, p.Ala3294Prof s*4	NM_003482.3
		Slavotinek (2024) [39]	Syndromic	MT‐TL1	m:3243A>G	NC_012920.1
		Slavotinek (2024) [39]	Isolated	MYRF	c.2074C>G, p.Leu692Val	NM_001127392.3
		Reuter (2020) [30]	Syndromic [global developmental delay, hypotonia, borderline short stature and gastroesophageal reflux]	POGZ	c.3403delG, p.(Glu1135Argfs*3)	(NM_015100.3)
		Alankarage (2019) [29]	Isolated	SMAD6	c.86del, p.Gly29Alafs*35	NM_005585.4
	BAV (*n* = 3)	Sweeney (2021) [32]	Syndromic [prolonged QT syndrome prolonged QTc syndrome pulmonary hypertension hip dysplasia congenital microcephaly feeding difficulties aspiration failure to thrive]	POLR1C, POLR1C	c.242T>C, p.Leu81Pro, c.326G>A, p.Arg109His	N/A
		Slavotinek (2024) [39]	Syndromic	TTPA	c.552G>A, p.Thr184 =	NM_000370.3
		Slavotinek (2024) [39]	Syndromic	ZEB2	c.2672_2678delCCAAACC	NM_014795.3
	Shone's (*n* = 3)	Sweeney (2021) [32]	Syndromic [small for gestational age congenital diaphragmatic hernia congenital heart disease hypoplasia of corpus callosum abnormal face shape recurrent respiratory infections delayed gross motor development]	ARID1B	c.3096_3100delCAAAG, p.Lys1033ArgfsTer32	N/A
		Reuter (2020) [30]	Syndromic [developmental delay (gross motor, speech and language), failure to thrive, tracheal tug on inspiration, high palate, triangular face, ptosis, hyperteloric, protruding forward facing ears, sacral dimple, eczema, and dysmorphisms]	KMT2D	c.15673C>T, p.(Arg5225Cys)	(NM_003482.3)
		Alankarage (2019) [29]	Isolated	TLL1	c.2578A>G, p.Thr860Ala	NM_012464.4
	AS (*n* = 2)	Slavotinek (2024) [39]	Syndromic	MED13	c.4198C>T, p.Arg1400Ter	NM_005121.2
		Reuter (2020) [30]	Syndromic [global developmental delay, self‐injurious behavior, microcephaly, short stature, hypotonia, joint laxity, and dysmorphic features]	NEXMIF	c.1502delG, p.(Gly501Valfs*4)	(NM_001008537.2)
	IAA	Slavotinek (2024) [39]	Syndromic	SCN4A	c.4364T>C, p.Ile1455Thr	NM_000334.4
	BAV/MS	Sweeney (2021) [32]	Syndromic [ankyloglossia aspiration, silent aspiration laryngomalacia subglottic stenosis pelviectasis, hydronephrosis]	CHD7	c.7879C>T, p.Arg2627Ter	N/A
Conotruncal lesions [27, 29, 30, 35, 37, 38, 39](*n* = 26)	TOF (*n* = 10)	Alankarage (2019) [29]	Syndromic [esophageal atresia, duodenal atresia, tracheoesophageal fistula, imperforate anus with mucous fistula, right multi‐cystic dysplastic kidney with bilateral hydronephrosis, severe penoscrotal hypospadias, vertebral defects, bilateral fixed talipes deformities]	ACVR2B, DOCK6	c.1057G>T, p.Gly353Trp, c.3163G>A, Val1055Met	NM_001106.3, NM_020812.3
		Hauser (2017) [27]	Syndromic [choanal atresia, hypoplasia of the semicircular canal]	CHD7	c.5051‐1G>A	NM_017780.3
		Hays (2023) [37]	Syndromic [colobomas, left microphthalmia, cleft lip and palate, cryptorchidism, micropenis, intraventricular hemorrhage]	CHD7	c.1879dup, p.Gln627Profs Ter5	NM_017780.4
		Slavotinek (2024) [39]	Syndromic	CHD7	c.4185+1G>A	NM_017780.4
		Reuter (2020) [30]	Syndromic [mild congenital lymphedema]	FLT4	c.89delC, p.(Pro30Argfs*3)	(NM_182925.4)
		Slavotinek (2024) [39]	Syndromic	GREB1L	c.157+1delG	NM_001142966.2
		Hauser (2017) [27]	Syndromic [jejunal atresia, microcolon preauricular skin tag, cholestasistriangular‐shaped face]	JAG1	c.551G>A, p.Arg184His	NM_000241.2
		Alankarage (2019) [29]	Isolated	NF1	c.3560T>G, p.Leu1187Arg	NM_000267.3
		Slavotinek (2024) [39]	Isolated	NKX2‐5	c.439del, p.Gln147 fs	NM_004387.4
		Slavotinek (2024) [39]	Syndromic	ZIC3	c.1103del, p.Arg368ProfsTer40	NM_003413.4
	Truncus (*n* = 8)	Slavotinek (2024) [39]	Isolated	DLL4	c.1857_1864del, p.Pro621 fs	NM_019074.4
		Yaoita (2024) [38]	Syndromic [shortening of the lingual pedicel, left migrating testis, left polydactyly]	GATA6	c.1367G>A, p.(Arg456His)	N/A
		Yaoita (2024) [38]	Isolated	NOTCH1	c.545G>A, p.(Cys182Tyr)	N/A
		Yaoita (2024) [38]	Isolated	TMEM260	c.1617del, p.Trp539Cysfs*7	N/A
		Yaoita (2024) [38]	Syndromic [posthemorrhagic hydrocephalus, enuresis, myopia, amblyopia, hearing impairment]	TMEM260	c.1617del, p.Trp539Cysfs*7	N/A
		Yaoita (2024) [38]	Isolated	TMEM260	c.1617del, p.Trp539Cysfs*7	N/A
		Yaoita (2024) [38]	Isolated	TMEM260, TMEM260	c.332dup, p.(Thr112Hisfs*36), c.1617del, p.Trp539Cysfs*7	N/A
		Yaoita (2024) [38]	Syndromic [hydronephrosis]	TMEM26, TMEM260	c.1617del, p.Trp539Cysfs*7, c.1960C>T, p.(Gln654*)	N/A
	DORV (*n* = 6)	Alankarage (2019) [29]	Isolated	CHD7	c.2098A>G, p.Asn700Asp	NM_017780.3
		Alankarage (2019) [29]	Isolated	JAG1	c.622G>C, p.Gly208Arg	NM_000214.2
		Hays (2023) [37]	Syndromic [pentalogy of cantrell]	MYH6	c.1138G>A, p.Glu380Lys	NM_002471.3
		Alankarage (2019) [29]	Isolated	NOTCH1	c.6105del, Ala2036Profs*3	NM_017617.4
		Alankarage (2019) [29]	Syndromic [right foot: 2nd 3rd toes, absent middle and distal phalanges; 4th toe, hypoplastic middle phalanges, absent distal phalanges; 5th toe, hypoplastic middle and distal phalanges. Left foot: 2nd and 3rd toes, hypoplastic middle]	NOTCH1	c.4416C>G, p.Cys1472Trp	NM_017617.4
		Hays (2023) [37]	Syndromic [refractive error, renal duplication]	PUF60	c.24+1G>A	NM_078480.2
	TGA (*n* = 2)	Blue (2022) [35]	Isolated	MYBPC3	c.2526C>G, p.Tyr842Ter	NM_000256.3
		Alankarage (2019) [29]	Isolated	NODAL	c.919C>T, p.Arg307	NM_018055.4
PDA [30, 39] (*n* = 14)	PDA (*n* = 14)	Slavotinek (2024) [39]	Syndromic	ARID1B	c.1293_1317del, p.Ala433 fs	NM_001374828.1
		Slavotinek (2024) [39]	Syndromic	BMP2	c.939G>A, p.Trp313Ter	NM_001200.4
		Slavotinek (2024) [39]	Syndromic	COL2A1	c.2594del, p.Pro865 fs	NM_001844.5
		Slavotinek (2024) [39]	Syndromic	ERF	c.517_521dup, p.Ala175 fs	NM_006494.4
		Slavotinek (2024) [39]	Syndromic	FAM111 A	c.931 A>T, p.Ile311Phe	NM_001312909.2
		Slavotinek (2024) [39]	Syndromic	GRIN2B	c.2045G>A, p.Arg682His	NM_000834.3
		Reuter (2020) [30]	Isolated	MYH11	c.4578+1G>A, p.?	(NM_002474.2)
		Slavotinek (2024) [39]	Syndromic	NF1, MADD	c.1845+1G>A, c.3070 C>T, p.Gln1024Ter	NM_000267.3, NM_003682.4
		Slavotinek (2024) [39]	Syndromic	PEX26	c.34dup, p.Leu12 fs	NM_001127649.3
		Slavotinek (2024) [39]	Syndromic	SCN1A	c.707T>C, p.Ile236Thr	NM_006920.5
		Slavotinek (2024) [39]	Syndromic	SETBP1	c.2608G>A, p.Gly870Ser	NM_015559.3
		Slavotinek (2024) [39]	Syndromic	SMC1A	c.3592G>A, p.Glu1198Lys	NM_006306.4
		Slavotinek (2024) [39]	Syndromic	TFAP2A	c.752A>G, p.Arg251Gly	NM_001042425.2
		Slavotinek (2024) [39]	Isolated	TMEM260	c.377C>T, p.Ala126Val	NM_017799.4
Right obstructive lesions [29, 32, 34, 36, 39] (*n* = 12)	PS (*n* = 9)	Cao (2022) [34]	Syndromic [suspected fetal IVH, bilateral lateral ventricle heterogeneity]	COL4A1	c.2281G>A, p.G761 R	NM_001845.6
		Sweeney (2021) [32]	Syndromic [short neck redundant skin in nuchal area mild retrognathia thrombocytopenia hyperthyroidism neonatal graves' disease]	KMT2D	c.3228_3230delGAA, p.Lys1077del	N/A
		Weaver (2022) [36]	Isolated	NOTCH1	c.4918del, A1640Hfs*9	N/A
		Cao (2022) [34]	Syndromic [polyhydramnios, cerebral ventriculomegaly]	PTPN11	c.1505C>T, p.S502 L	NM_002834.4
		Weaver (2022) [36]	Isolated	PTPN11	c.1507G>A, G503 R	N/A
		Weaver (2022) [36]	Isolated	PTPN11	c.922A>G, N308D	N/A
		Slavotinek (2024) [39]	Syndromic	SHOC2	c.4A>G, p.Ser2Gly	NM_007373.3
		Slavotinek (2024) [39]	Syndromic	TGFBI	c.1663C>T, p.Arg555Trp	NM_000358.3
		Sweeney (2021) [32]	Syndromic [aganglionic megacolon generalized hypotonia coronal hypospadias abnormality of corpus callosum abnormality of philtrum]	ZEB2	c.656delG, p.Gly219AlafsTer5	N/A
	Tricuspid atresia	Slavotinek (2024) [39]	Syndromic	RFXANK	c.454_455del, p.Ile152 fs	NM_003721.4
	Hypoplastic right heart	Alankarage (2019) [29]	Syndromic [autism, chylous effusion, polyneuropathy, seasonal asthma]	NOTCH1	c.5865del, p.Asn1955Lysfs*26	NM_017617.4
	PA/IVS	Sweeney (2021) [32]	Isolated	NF1, MYBPC3	c.5118delT, p.Val1707PhefsTer3, c.3184delG, p.Val1062LeufsTer13	N/A
Not specified [40] (*n* = 12)		Austin (2024) [40]	Syndromic	CHD7	c.6883_6884insA, p.(Ser2295Tyrfs*8)	NM_017780.2
		Austin (2024) [40]	Syndromic	CHD7	c.5405‐17G>A,	NM_017780.2
		Austin (2024) [40]	Isolated	CDK13	c.484dupG, p.(Ala357Serfs*11)	NM_003718.4
		Austin (2024) [40]	Isolated	ELN	c.1069_1091del, p.(Ala357Serfs*11)	NM_000501.3
		Austin (2024) [40]	Isolated	FLT4	c.1755 C>G, p.(Tyr585*)	NM_182925.4
		Austin (2024) [40]	Isolated	GATA4	c.851_853delGCA, p.(Arg284_Asn285delinsHis)	NM_002052.4
		Austin (2024) [40]	Isolated	JAG1	c.1823_1826del, p.(Gln608Argfs*134)	NM_000214.3
		Austin (2024) [40]	Isolated	JAG1	c.1181dupA, p.(Asn394Lysfs*4)	NM_000214.3
		Austin (2024) [40]	Isolated	NKX2	c.487delC, p.(Leu163Cysfs*13)	NM_004387.3
		Austin (2024) [40]	Isolated	NODAL	c.158_165del, p.(Pro53Argfs*23)	NM_018055.5
		Austin (2024) [40]	Isolated	NOTCH1	c.5496_5505delinsGGT, p.(Asp1833Valfs*52)	NM_017617.5
		Austin (2024) [40]	Isolated	PTPN11	c.236 A>G, p.(Gln79Arg)	NM_002834.3
Other [30, 32, 34, 39] (*n* = 10)	Dextrocardia (*n* = 3)	Cao (2022) [34]	Syndromic [situs inversus]	DNAH5, DNAH5	c.5563dupA, p.I1855 fs, c.6442C>T, p.Q2148X	NM_001369.2, NM_001369.2
		Slavotinek (2024) [39]	Syndromic	DNAH5	c.13486C>T, p.Arg4496Ter	NM_001369.2
		Slavotinek (2024) [39]	Syndromic	MYRF	c.1200C>A, p.Asn400Lys	NM_001127392.3
	PPS (*n* = 2)	Slavotinek (2024) [39]	Isolated	JAG1	c.588C>A, p.Cys196Ter	NM_000214.3
		Slavotinek (2024) [39]	Syndromic	NADYSN1	c.1459C>T, p.Arg487Ter	NM_018161.5
	Right arch	Slavotinek (2024) [39]	Syndromic	BRPF1	c.1755_1756delinsT, p.Lys585 fs	NM_001003694.2
	Dysplastic TV	Slavotinek (2024) [39]	Syndromic	MYOD1	c.188C>A, p.Ser63Ter	NM_002478.5
	PS, dysplastic AoV	Slavotinek (2024) [39]	Syndromic	PTPN11	c.184T>G, p.Tyr62Asp	NM_002834.5
	PS, AI	Reuter (2020) [30]	Isolated	PTPN11	c.209A>G, p.(Lys70Arg)	(NM_002834.4)
	Rhabdomyoma	Sweeney (2021) [32]	Syndromic [hamartoma of the eye cerebral hamartomas]	TSC2	c.935_936delTC, p.Leu312GlnfsTer25	N/A
Single ventricle [29, 30, 32, 39] (*n* = 5)	Single ventricle (*n* = 2)	Alankarage (2019) [29]	Isolated	GATA6	c.1595_1596del, p.Pro532Hisfs*100	NM_005257.5
		Alankarage (2019) [29]	Isolated	NODAL	c.123_142dup, Tyr48Trpfs*5	NM_018055.4
	Mitral atresia, DORV, coarctation	Slavotinek (2024) [39]	Syndromic	ARID1A	c.750_771dup, p.Ser258 fs	NM_006015.6
	Unbalanced AVSD	Reuter (2020) [30]	Syndromic [borderline microcephaly, global developmental delay, hypotonia]	NIPBL	c.771+1G>A, p.?	(NM_133433.3)
	Hypoplastic LV	Sweeney (2021) [32]	Syndromic [hydrops fetalis fetal ascites pericardial effusion]	TPM1	c.533G>A, p.Arg178His	N/A
Heterotaxy [29, 37, 39] (*n* = 5)	Heterotaxy, single ventricle (*n* = 2)	Alankarage (2019) [29]	Syndromic [asplenia]	NODAL	c.123_142dup, p.Tyr48Trpfs*5	NM_018055.4
		Hays (2023) [37]	Syndromic [craniosynostosis, external ear malformation, ventriculomegaly, heterotaxy]	PTPN11	c.179G>C, p.Gly60Ala	NM_002834.4
	Heterotaxy, dextrocardia	Slavotinek (2024) [39]	Syndromic	DNAH5, DNAH5	c.10815del, p.Pro3606 fs, c.11028+5G>A	NM_001369.3, NM_001369.3
	Heterotaxy, TAPVR	Hays (2023) [39]	Syndromic [fetal growth restriction, heterotaxy]	FOXJ1	c.867dup, p.Ser290Glnfs Ter12	NM_001454.4
	Heterotaxy, unbalanced AVSD	Alankarage (2019) [29]	Isolated	INVS	c.3182dup, Asn1061Lysfs*20	NM_014425.4
Venous abnormality [32, 34] (*n* = 2)	Systemic vein variant (retroesophageal left brachiocephalic vein merged into azygos vein)	Cao (2022) [34]	Syndromic [dilated bilateral renal pelvis, and overgrowth]	KRAS	c.178G> A, p.G60S	NM_004985.5
	PAPVR	Sweeney (2021) [32]	Isolated	FOXF1	c.188G>T, p.Ser63Ile	N/A

Abbreviations: AI, aortic insufficiency; AoV, aortic valve; ASD, atrial septal defect; AVSD, atrioventricular septal defect; BAV, bicuspid aortic valve; CHD, congenital heart disease; DORV, double outlet right ventricle; HLHS, hypoplastic left heart syndrome; IAA, interrupted aortic arch; IVH, Intraventricular Hemorrhage; LV, left ventricle; MS, mitral stenosis; PA/IVS, Pulmonary Atresia with Intact Ventricular Septum; PAPVR, partial anomalous pulmonary venous return; PDA, patent ductus arteriosus; PPS, peripheral pulmonary stenosis; PS, pulmonary stenosis; TAPVR, total anomalous pulmonary venous return; TGA, transposition of the great arteries; TOF, tetralogy of Fallot; TV, tricuspid valve; VSD, ventricular septal defect.

**TABLE 4:** Yield of whole genome sequencing by classification type for pathogenic/likely pathogenic variants.

Classification	Studies (n)	WGS positive (n)	Total WGS (n)	Pooled proportion % (95% CI)
All cases	14 [27, 28, 29, 30, 31, 32, 33, 34, 35, 36, 37, 38, 39, 40]	165	933	17.83% (10.20%–26.78%)
Isolated CHD^a^	10 [27, 29, 30, 32, 34, 35, 37, 38, 39, 40]	38	418	9.83% (0.51%–25.20%)
Syndromic CHD^b^	11 [27, 29, 31, 32, 33, 34, 35, 37, 38, 39, 40]	117	444	22.36% (13.26%–32.27%)
CHD with extracardiac anomalies^c^	11 [27, 29, 31, 32, 33, 34, 35, 37, 38, 39, 40]	115	441	21.66% (12.60%–32.08%)

Abbreviations: CHD, congenital heart disease; CI, confidence interval; WGS, whole genome sequencing.

^a^ Isolated CHD: No other abnormalities.

^b^ Syndromic CHD: Presence of other structural anomalies, developmental abnormalities, or distinctive features.

^c^ CHD with extracardiac anomalies: Based on structural findings alone not accounting developmental abnormalities (was used as a proxy for the classification possible prenatally where only structural anomalies and not developmental outcomes can be assessed).

## 3 Results

**FIGURE 1:**
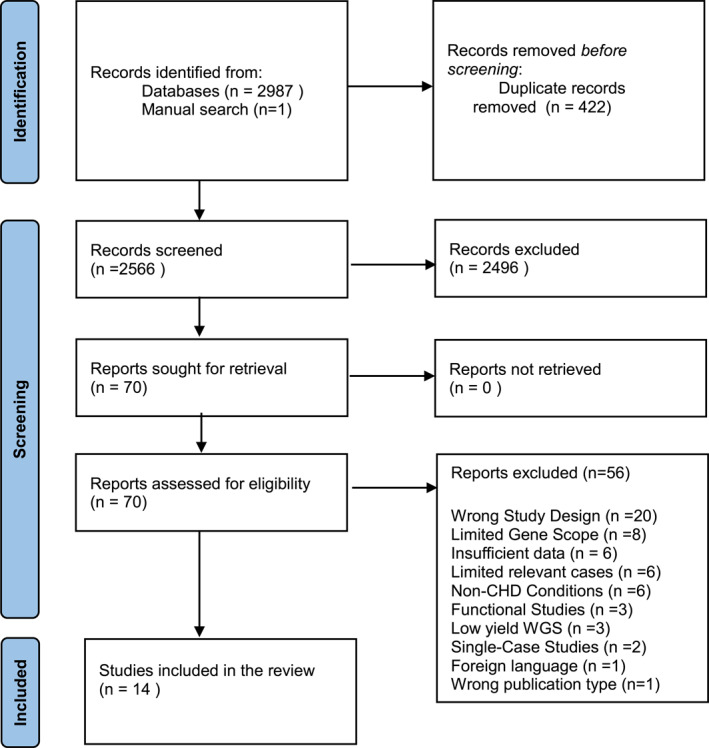
PRISMA flow chart.

The PRISMA flowchart (Figure 1) outlines the systematic selection process for this review. A total of 2987 records were identified from database searches, and one article was identified through manual searches, with 422 duplicates removed, leaving 2566 records for screening. Of these, 2496 were excluded from irrelevance, resulting in 70 reports sought for retrieval, all of which were successfully accessed. After assessing eligibility, 56 reports were excluded due to reasons such as wrong study design, limited gene scope, insufficient data, or irrelevance to CHD. Excluded studies and reasons for exclusion can be found in Supporting Information S1. Ultimately, 14 studies met the inclusion criteria, comprising a total of 933 CHD cases, of which 165 had P/LP SNVs.

### 3.1 Study Characteristics

Table 1 outlines the study characteristics. The systematic review included 14 studies [27, 28, 29, 30, 31, 32, 33, 34, 35, 36, 37, 38, 39, 40] from diverse geographic regions, including seven studies from the USA, three from China, two from Australia, and one from Canada and Japan. Nine studies utilized a prospective design, and five were retrospective. Inclusion criteria varied but generally targeted neonatal and pediatric patients with CHD of unknown genetic etiology. Ten studies included only the CHD population, while three included a population with various structural anomalies. Two studies assessed patients prenatally [28, 34], and the rest were postnatal. Most used ACMG criteria and validated variants with Sanger sequencing, indicating a low risk of bias. The age at follow‐up varied across the studies.

### 3.2 Phenotype Associations by Gene in P/LP SNVs

Table 2 outlines several key genes in which P/LP variants were reported among CHD cases. These genes were observed in cases with either syndromic or isolated phenotypes, reflecting the potential genetic heterogeneity of CHD.

- *CHD7 (n = 13)*: Definitive for CHARGE syndrome, CHD7 was the most frequently implicated gene across the included studies, with 13 cases reported in 7 studies. In six studies, CHD7 variants were syndromic. In the available cases, the most common cardiac diagnoses were conotruncal defects, left obstructive lesions, or septal defects. Variants such as c.5051‐1G>A [27], c.1879dup, and p.Gln627ProfsTer5 [37] were reported in cases with severe syndromic phenotypes.
- *PTPN11 (n = 10)*: Recognized as the most commonly implicated gene in Noonan syndrome, PTPN11 has been reported in both isolated (4 cases) [30, 36, 40] and syndromic (6 cases) [30, 34, 37, 39] CHD. Among isolated cases, CHD typically presented as right‐sided obstructive lesions such as PS or as aortic insufficiency (AI). Syndromic CHD included right‐sided, left‐sided, or both left and right‐sided lesions.
- *TMEM260 (n = 7)*: Among the 7 reported cases, TMEM260 variants were predominantly observed in conotruncal lesions, with recurrent changes like c.1617del in the Japanese population [38]. Syndromic features reported alongside TMEM260 variants included hydrocephalus, hearing impairments, and hydronephrosis.
- *JAG1 (n = 6)*: Variants in this gene were observed with both isolated and syndromic features and CHD types reported included conotruncal lesions and septal defects [27, 29, 39, 40].
- *NOTCH1 (n = 6)*: In cases harboring NOTCH1 variants, CHD manifestations included right‐sided obstructive lesions [29, 36] and conotruncal lesions [29, 38].
- *KMT2D (n = 5)*: As the definitive gene in Kabuki syndrome, variants of this gene have been reported in a range of CDH phenotypes, including left‐sided obstructive lesions [30, 37, 39], right‐sided obstructive lesions [32], and ventricular septal defect (VSD) [39].
- Other Genes: Additional genes were observed in a small number of cases, including **NODAL (n = 4)** (single ventricle and conotruncal lesion) [29, 40], **DNAH5 (n = 3)** (dextrocardia) [34, 39], **GATA6 (n = 3)** (conotruncal lesion, single ventricle, and VSD) [29, 38, 39], **NF1 (n = 3)** (TOF, PDA, and PA/IVS) [29, 32, 39]. There were two cases of each of the following genes with a mix of both isolated and syndromic phenotypes: **ARID1B, MYH6, COL2A1, FOXF1, GATA4, GREB1L, MYBPC3, MYRF, ZEB2, FLT4.** The rest of the reported genes (*n* = 82) appeared in one case.

### 3.3 Phenotype Associations by CHD in P/LP SNVs

Table 3 outlines the classification of CHD phenotypes into specific categories.

- *Septal Defects (n = 37)* [29, 30, 39]: These were the most common CHD phenotype, including VSD (*n* = 18), ASD (*n* = 14), and AVSD (*n* = 5). The most common genes reported in these cases were CHD7, GATA4, and KMT2D. All septal defects occurred in cases that were reported as syndromic.
- *Left‐sided Obstructive Lesions (n = 35)* [29, 30, 32, 33, 37]: The most common lesions were coarctation of the aorta (*n* = 16), HLHS (*n* = 9), BAV (*n* = 3), and Shone's (*n* = 3), with reported genes including CHD7, KMT2D, and PTPN11.
- *Conotruncal Lesions (n = 26)* [27, 29, 30, 35, 37, 38]: The most common lesions were TOF (*n* = 10), truncus arteriosus (*n* = 8), DORV (*n* = 6), and TGA (*n* = 2). The most common genes reported in these cases included CDH7, TMEM260 (both isolated and syndromic forms of TA), and NOTCH1.
- *PDA (n = 14)* [30, 39]: Among the cases, only one was an isolated finding associated with an SNV TMEM260.
- *Right‐sided obstructive lesions (n = 12)* [29, 32, 34, 36, 39]: included PS (*n* = 9) and one case of TA, HRH, and PA with intact ventricular septum. PTPN11 variants were reported in several of these cases.
- Other CDH phenotypes [30, 32, 34, 39] were **single ventricle** (*n* = 5 ARID1A, GATA6, NIPBL, NODAL, and PTPN11), **heterotaxy** (*n* = 5, two cases due to DNAH5), and **venous abnormalities** (*n* = 2, KRAS and FOXF1). In 12 cases, CHD type was unspecified, and 10 were classified as other (Table 3).

### 3.4 Meta‐Analysis of Pooled Proportions of WGS Yield in P/LP SNVs

The pooled proportion of P/LP for all CHD cases combined was 17.83% (95% CI: 10.20%–26.78%) across 14 studies, with 165 positive WGS results out of 933 total cases (Supporting Information (Figure S1)). Isolated CHD had a pooled proportion of 9.83% (95% CI: 0.51%–25.20%) from 10 studies, with 38 positive results out of 418 cases (Supporting Information (Figure S2)). CHD with extracardiac anomalies showed a higher yield, with a pooled proportion of 21.66% (95% CI: 12.60%–32.08%) from 11 studies, with 115 positive results out of 441 cases (Supporting Information (Figure S3)). Syndromic CHD demonstrated the highest yield, with a pooled proportion of 22.36% (95% CI: 13.26%–32.27%) from 11 studies, with 117 positive results out of 444 cases (Supporting Information (Figure S4)) (Table 4).

Twenty out of 105 cases across four studies had negative CMA followed by positive WGS, resulting in an incremental yield of 20% (95% CI: 8.00%–34.00%) (Supporting Information (Figure S5)).

### 3.5 Variants of Uncertain Significance

Supporting Information S1 provides a detailed overview of cases with VUS, focusing on genes with established associations according to the ClinGen Gene‐Disease Validity Classification. Out of the total cases, 41 were found in genes classified by the ClinGen Congenital Heart Disease Expert Curation Panel to have a CHD association. The most frequently reported gene was MYH6, with seven cases involving various CHD types such as septal defects, conotruncal lesions, and other anomalies. Other significant genes included GATA6, GATA4, and NKX2‐5, each linked to multiple CHD presentations. CHD types ranged from septal defects and left obstructive lesions to complex conotruncal anomalies such as TOF and TGA. In contrast, 22 cases were in genes not classified by ClinGen to have a CHD association.

### 3.6 Quality Assessment

**FIGURE 2:**
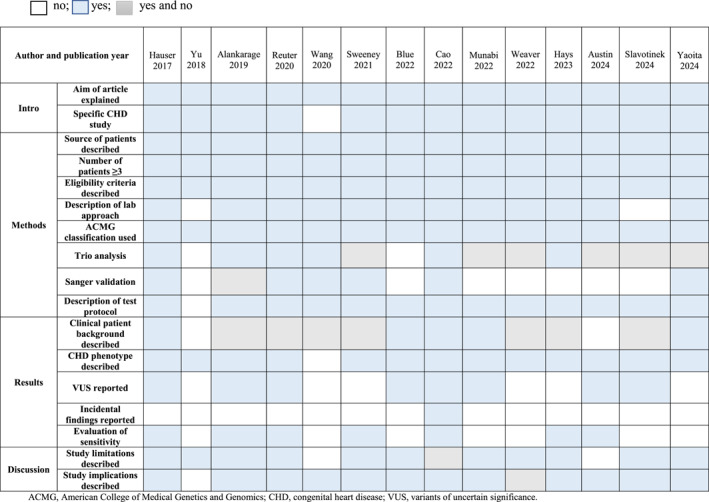
Quality assessment using modified Standards for Reporting of Diagnostic Accuracy (STARD).

The quality assessment of the studies using modified STARD criteria shows high reporting standards (Figure 2). Most studies clearly stated their aims and specified the CHD type. Methods were well‐documented, including patient sources, eligibility criteria, and lab approaches with ACMG classification and trio analysis. Sanger validation was performed in six of the 13 studies that identified variants, whereas seven did not report their validation method, indicating a moderate risk of bias in variant validation. Results sections detail clinical backgrounds and CHD phenotypes, with many studies reporting VUS and incidental findings. Sensitivity evaluations were less consistent. Discussions typically included study limitations and implications, highlighting the methodological rigor and transparency.

## 4 Discussion

### 4.1 Summary of Principal Findings

This systematic review and meta‐analysis evaluated the diagnostic yield of WGS in CHD for P/LP SNVs. The pooled diagnostic yield was 17.83% (95% CI: 10.20%–26.78%), with syndromic CHD cases demonstrating the highest yield at 22.36%. Structural CHD with extracardiac anomalies was followed with a yield of 21.66%, while isolated structural CHD had the lowest diagnostic rate at 9.83%. Several genes **CHD7**, **PTPN11**, and **TMEM260** have been recurrently reported in CHD phenotypes.

### 4.2 Results in the Context of What Is Known

WGS builds upon methods like CMA and WES, offering a more comprehensive view by capturing both coding and non‐coding regions as well as CNVs in a single assay. Previous studies have shown that CMA has a diagnostic yield of 10%–15% for CHD, with limited ability to detect small indels, and does not assess for SNVs [41, 42, 43]. WES, which focuses on coding regions, typically yields an incremental diagnostic yield over CMA of 17.4% for all prenatally diagnosed CHDs, 9.3% for isolated CHDs, and 25.9% for CHDs with extracardiac anomalies according to 2024 systematic review [44]. While our study is the first systematic review focusing on the yield of WGS to detect LP/P SNVs in CHD, a 2024 systematic review that included various structural anomalies in the prenatal period and infancy showed that the incremental yield of WGS over CMA was 26%, 16%, and 39% for all prenatal and postnatal cases, respectively. The incremental yield of WGS over CMA and WES was 1% [45]. Another 2023 systematic review showed a higher diagnostic yield of WGS for pediatric patients with suspected genetic disorders than for WES (OR = 1.54, 95% CI 1.11–2.12) [46].

By covering the entire genome, WGS improves upon these methods. This is particularly relevant in CHD, where variants in non‐coding regions may disrupt promoters, enhancers, or splicing mechanisms, as seen with **CHD7** in CHARGE syndrome and **PTPN11** in Noonan syndrome [47]. Rapid WGS (rWGS) has further demonstrated its utility, as shown by Sweeney 2024, who reported a 46% diagnostic yield in 24 CHD infants using rWGS compared to 10% with CMA and gene panels (*p* = 0.02) [32]. Similarly, Hays 2023 found that rWGS diagnosed genetic disorders in 27% of 48 cases of critically ill infants with CHD and led to management changes in 62% of diagnostic cases [37].

Despite its advantages, barriers to WGS implementation persist. High costs, extended turnaround times, and limited access in underserved regions hinder its widespread adoption [48]. However, Sweeney 2024 demonstrated that rWGS reduced hospital spending post‐diagnosis, highlighting its cost‐effectiveness in acute care settings. Furthermore, timely molecular diagnoses from rWGS provided actionable insights influencing clinical decisions and resource utilization [32]. Limitations are even more complex in prenatal cases. The high number of VUS cases and variant interpretation without observable phenotype and developmental milestones makes it challenging to reach a definitive diagnosis, resulting in uncertainty and distress for parents [49, 50]. Timely decision‐making is another important factor for cases detected in late pregnancy, which can limit its utility [51]. Current cost and limited insurance coverage may also restrict its use and should be reviewed before ordering the testing.

Existing guidelines for the use of WGS and WES emphasize their application, particularly when other diagnostic methods have failed or are less practical.

The American College of Obstetricians and Gynecologists (ACOG) and the Society for Maternal‐Fetal Medicine (SMFM) do not recommend the routine use of WGS or WES for prenatal diagnosis outside of clinical trials. However, in select circumstances, such as recurrent or lethal fetal anomalies, WES may be considered [52]. Other societies, such as the International Society of Prenatal Diagnosis (ISPD), are starting to accept WES and WGS as routine components of prenatal testing. Guidelines and points of consideration have now been published to help guide the data‐driven and equitable implementation of these tests [53].

The American College of Medical Genetics and Genomics (ACMG) and the American Academy of Pediatrics (AAP) recommend WES and WGS for children with complex medical conditions such as unexplained developmental delays, intellectual disabilities, or multiple congenital anomalies that are suspected to have a genetic basis [54, 55, 56].

### 4.3 Strengths and Limitations

Rigorous methodology, use of standardized variant classification, and diverse populations are the strengths of this review. Subgroup analyses provided nuanced insights into diagnostic yields across syndromic, isolated, and extracardiac CHD cases, informing clinical practice. Our re‐classification of all cases into one of two categories, either structural CHD only or structural CHD with other extra‐cardiac structural anomalies, is a proxy for diagnostic yield in the prenatal setting when information about neurodevelopmental status and detailed dysmorphology exam findings are not yet available.

However, there are several considerable limitations. Given that the majority of included studies were conducted postnatally, our analysis may underrepresent severe CHD cases diagnosed prenatally that did not progress to live birth due to spontaneous miscarriage, elective termination, or intrauterine demise. Differences in follow‐up durations may lead to missed syndromic diagnoses, as some traits appear later. Heterogeneity arising from differences in sequencing platforms, population demographics, and phenotypic classification systems may have introduced bias.

### 4.4 Future Directions

Prospective, multicenter study designs with standardized, broad inclusion criteria should be utilized in future studies to decrease the selection of cases with severe or syndromic phenotypes and therefore minimize selection bias. Quality and consistency of phenotypic data, standardized reporting, and development of a core outcome set are also important. In addition, a recent publication has reported a higher probability of genetic diagnosis, particularly aneuploidies in CHD cases associated with fetal growth restriction (FGR) [57]. Use of WGS in CHD cases in the context of additional findings such as FGR may enhance the identification of high‐risk pregnancies and guide more targeted genomic testing strategies.

## 5 Conclusions

Our findings highlight the increasing value of WGS in CHD diagnostics, with a pooled diagnostic yield of 17.83%, 22.36%, and 9.83% in all CHDs, syndromic, and isolated CHD, respectively. WGS offers a higher yield in syndromic and complex CHD and may be most effective when used as an early diagnostic tool in these cases. For isolated CHD, a stepwise approach may be more appropriate.

## Ethics Statement

The authors have nothing to report.

## Consent

The authors have nothing to report.

## Conflicts of Interest

The authors declare no conflicts of interest.

## Supporting information


Supporting Information S1

**Figure S1**: Yield of whole genome sequencing in all CHD cases.

**Figure S2**: Yield of whole genome sequencing in isolated CHD cases.

**Figure S3**: Yield of whole genome sequencing in CHD with extracardiac abnormalities not accounting for developmental abnormalities.

**Figure S4**: Yield of whole genome sequencing in syndromic CHD cases.

**Figure S5**: Incremental yield of whole genome sequencing over CMA.

## Data Availability

The data that support the findings of this study are available from the corresponding author upon reasonable request.
