# Supplementary material for: Yield of Whole Genome Sequencing for Pathogenic Single Nucleotide Variants in Congenital Heart Disease: A Systematic Review and Meta‐Analysis
Source: Prenat Diagn. 2025 Sep 4;46(5-6):780–818. doi: 10.1002/pd.6878 (PMC13170038; doi:10.1002/pd.6878)
Supplement: Supplementary file 1 — Supporting Information S1 [file PD-46-780-s004.docx]

**Supplement 1:** Search Strategy - **search date August 26^th^, 2024**

| **Set** | **PubMed** | **Results** |
| --- | --- | --- |
| #1 | (whole genome sequencing) AND (whole exome sequencing OR chromosomal microarray analysis) | 14,546 |
| #2 | "heart defects, congenital"[MeSH Terms] OR ("Heart"[All Fields] AND ("anomal*"[All Fields] OR "abnormal*"[All Fields] OR "defect*"[All Fields] OR "disease*"[All Fields] OR "malform*"[All Fields] OR "deform*"[All Fields]) AND ("congenital*"[All Fields] OR "fetal*"[All Fields] OR "birth*"[All Fields])) | 223,289 |
| #3 | #1 AND #2 | **405** |
| **Set** | **Cochrane Library** | **Results** |
| #1 |  | **0** |
| **Set** | **Scopus** | **Results** |
| #1 | TITLE-ABS-KEY ( ( ( whole OR complete ) AND ( ( gen* ) AND ( sequenc* ) ) ) AND ( ( ( whole OR complete ) AND ( ( gen* ) AND ( sequenc* ) ) ) OR ( chromosom* AND microarray AND analys* ) ) ) | 293,085 |
| #2 | TITLE-ABS-KEY ( "heart" AND ( "anomal*" OR "abnormal*" OR "defect*" OR "disease*" OR "malform*" OR "deform*" ) AND ( "congenital*" OR "fetal*" OR "birth*" ) ) | 177,942 |
| #3 | #1 AND #2 | **2,159** |
| **Set** | **Web of Science** | **Results** |
| #1 | ALL=((((whole OR complete) AND genome AND sequencing) AND (((whole OR complete) AND exome AND sequencing)) OR (chromosomal AND microarray AND analysis))) | 13,366 |
| #2 | ALL=(( "heart" AND ( "anomal*" OR "abnormal*" OR "defect*" OR "disease*" OR "malform*" OR "deform*" ) AND ( "congenital*" OR "fetal*" OR "birth*" ) )) | 93,549 |
| #3 | #1 AND #2 AND #3 | **423** |

**Supplement 2:** Excluded Studies

| **Author and year of publication** | **Reason for exclusion** |
| --- | --- |
| Rinne (2022)^1^ | Wrong Study Design |
| Calpena (2019)^2^ | Wrong Study Design |
| Zhao (2023)^3^ | Wrong Study Design |
| Baker (2022)^4^ | Wrong Study Design |
| Zhu (2021)^5^ | Wrong Study Design |
| Miller (2021)^6^ | Wrong Study Design |
| Theis (2020)^7^ | Wrong Study Design |
| Manshaei (2020)^8^ | Wrong Study Design |
| Birker (2023)^9^ | Wrong Study Design |
| Liu (2019)^10^ | Wrong Study Design |
| Richter 2020^11^ | Wrong Study Design |
| Stittrich (2014)^12^ | Wrong Study Design |
| Theis (2023)^13^ | Wrong Study Design |
| Theis (2022)^14^ | Wrong Study Design |
| Theis (2021)^15^ | Wrong Study Design |
| Wang (2021)^16^ | Wrong study design |
| Grunert (2020)^17^ | Wrong study design |
| Trevino (2020)^18^ | Wrong study design |
| Theis (2015)^19^ | Wrong study design |
| Wang (2016)^20^ | Wrong study design |
| Robinson (2020)^21^ | Limited Gene Scope |
| Cheng (2018)^22^ | Limited Gene Scope |
| Gu (2018)^23^ | Limited Gene Scope |
| Johnston (2018)^24^ | Limited Gene Scope |
| Bjornsson (2018)^25^ | Limited Gene Scope |
| Reuter (2019)^26^ | Limited Gene Scope |
| Qiao (2017)^27^ | Limited Gene Scope |
| Souza da Costa (2024)^28^ | Limited Gene Scope |
| Allred (2023)^29^ | Insufficient data |
| Helm (2024)^30^ | Insufficient data |
| Wu (2021)^31^ | Insufficient data |
| Owen (2023)^32^ | Insufficient data |
| Chen (2019)^33^ | Insufficient data |
| Chang (2023)^34^ | Insufficient data |
| Rosenhahn (2022)^35^ | Non-CHD Conditions |
| Du (2022)^36^ | Non-CHD Conditions |
| Chan (2016)^37^ | Non-CHD Conditions |
| Holt (2019)^38^ | Non-CHD Conditions |
| Tripolszki (2021)^39^ | Non-CHD Conditions |
| Goos (2017)^40^ | Non-CHD Conditions |
| Zhou (2023)^41^ | Limited relevant cases |
| Miceikaite (2023)^42^ | Limited relevant cases |
| Acevedo (2017)^43^ | Limited relevant cases |
| Hildebrandt (2021)^44^ | Limited relevant cases |
| Chung (2015)^45^ | Limited relevant cases |
| Nash (2019)^46^ | Limited relevant cases |
| Hrstka (2017)^47^ | Functional Studies |
| Xiao (2024)^48^ | Functional Studies |
| Theis (2015)^49^ | Functional Studies |
| Tan (2022)^50^ | Low yield WGS |
| Zhou (2021)^51^ | Low yield WGS |
| Xu (2018)^52^ | Low yield WGS |
| Wang (2016)^53^ | Single-Case Studies |
| Jia (2023)^54^ | Single-Case Studies |
| Gu (2020)^55^ | Foreign language |
| Sweeny (2021)^56^ | Wrong publication type (author correction) |

**Supplement 3**: Cases with Variants of Uncertain Significance

| **Author and year of publication** | **CHD type, main CHD diagnosis** | **Isolated or Syndromic** | **Gene (ClinGen Gene-Disease Validity Classification when available)** | **Variant** | **Inheritance** | **NM** |
| --- | --- | --- | --- | --- | --- | --- |
| Hauser (2017)^57^ | Septal defect, ASD | Isolated | MYH6 (definitive) | c.899-2A>G | Maternal | NM_002471.3 |
| Hauser (2017)^57^ | Left obstructive lesions, HLHS | Isolated | FOXL1 (disputed) | c.926A>G, p.Asn309Ser | Paternal | NM_005250.2 |
| Hauser (2017)^57^ | Left obstructive lesions, Coarctation | Syndromic [Multicystic kidney disease on left, hypoplastic kidney on right, infant of a diabetic mother, large for gestational age] | GATA4 (definitive) | c.1037C>T, p.Ala346Val | Maternal | NM_002052.3 |
| Hauser (2017)^57^ | Conotruncal lesion, TOF | Isolated | MYH6 (definitive) | c.C4768A, (p.Arg1590Ser) | Paternal | NM_002471.3 |
| Hauser (2017)^57^ | Left obstructive lesions, HLHS | Isolated | NKX2-5 (definitive) | c.C494T, p.Ala165Val | Paternal | NM_004387.3 |
| Hauser (2017)^57^ | Other, Ebstein's anomaly | Isolated | MYH7 (limited) | c.722C>T, p.Ser241Phe | Paternal | NM_000257.2 |
| Hauser (2017)^57^ | Heterotaxy, TGA | Syndromic [Preauricular skin tag, heterotaxy] | GATA6 (definitive) | c.C15G, p.Asp5Glu | Paternal | NM_005257.4 |
| Alankarage (2019)^58^ | Septal Defect | Syndromic | SOS1 (definitive) | c.3709C>A, Pro1237Thr | AD | NM_005633.3 |
| Alankarage (2019)^58^ | Not specified | Isolated | CRELD1 (limited) | c.1258A>G, Arg420Gly | AD | NM_015513.4 |
| Alankarage (2019)^58^ | Septal Defect | Syndromic | TLL1 (limited) | c.950G>A, Arg317His | AD | NM_012464.4 |
| Reuter (2020)^61^ | Conotruncal lesion, TOF | Syndromic [ADHD, autism, OCD, learning disability, psychiatric disorder] | GATA6 (definitive) | c.1302+123C>T | Maternal | NM_005257.4 |
| Reuter (2020)^61^ | Conotruncal lesion, TOF | Syndromic [Motor delay, short stature (<3rd centile)] | CRELD1 (limited) | c.610C>T, p.(Arg204Cys) | Paternal | NM_001031717.4 |
| Reuter (2020)^61^ | Conotruncal lesion, TOF | Isolated | GATA6 (definitive) | c.967T>C, p.(Tyr323His) | Paternal | NM_005257.5 |
| Reuter (2020)^61^ | Conotruncal lesion, TOF | Isolated | FLT4 (definitive) | c.401-9T>A, p.? | Maternal | NM_182925.4 |
| Reuter (2020)^61^ | Conotruncal lesion, TOF | Isolated | FLT4 (definitive) | c.2305G>A, p.(Glu769Lys) | Maternal | NM_182925.4 |
| Reuter (2020)^61^ | Right obstructive lesions, PA/IVS | Syndromic [Specific learning disability, atonic seizures, stroke post cardiac surgery] | TLL1 (limited) | c.160T>A, p.(Cys54Ser) | Maternal | NM_012464.5 |
| Reuter (2020)^61^ | Conotruncal lesion, Truncus | Syndromic [Minor dysmorphisms] | MYH6 (definitive) | c.2615G>A, p.(Arg872His) | Paternal | NM_002471.3 |
| Reuter (2020)^61^ | Conotruncal lesion, TGA | Isolated | PTPN11 (definitive) | c.661A>G, p.(Ile221Val) | Paternal | NM_002834.4 |
| Reuter (2020)^61^ | Conotruncal lesion, TGA | Syndromic [IUGR, abnormal hair whorls] | SOS2 (definitive) | c.622G>T, p.(Ala208Ser) |  | NM_006939.4 |
| Blue (2022)^59^ | Conotruncal lesion, TGA | Syndromic [multiple organ dysfunction syndrome] | RIT1 (definitive) | C650G, p.S217X |  | NM_006912 |
| Blue (2022)^59^ | Conotruncal lesion, TGA | Isolated | MYH6 (definitive) | c.A1226G, p.N409S |  | NM_002471 |
| Blue (2022)^59^ | Conotruncal lesion, TGA | Isolated | MESP1 (moderate) | c.C553T, p.Q185X |  | NM_018670 |
| Blue (2022)^59^ | Conotruncal lesion, TGA | Isolated | MYH6 (definitive) | c.T1673G, p.L558R |  | NM_002471 |
| Blue (2022)^59^ | Conotruncal lesion, TGA | Isolated | MED13L (limited) | c.C5360G, p.P1787R |  | NM_015335 |
| Blue (2022)^59^ | Conotruncal lesion, TGA | Isolated | MED13L (limited) | c.2013-2A>T |  | NM_015335 |
| Blue (2022)^59^ | Conotruncal lesion, TGA | Isolated | MED13L (limited) | c.2013-2A>T |  | NM_015335 |
| Blue (2022)^59^ | Conotruncal lesion, TGA | Isolated | MED13L (limited) | c.2013-2A>T |  | NM_015335 |
| Blue (2022)^59^ | Conotruncal lesion, TGA | Isolated | GJA1 (limited) | c.1127G>A, p.Arg376Gln |  | NM_000165.5 |
| Blue (2022)^59^ | Conotruncal lesion, TGA | Isolated | MYH7 (limited) | c.5459G>A, p.Arg1820Gln |  | NM_000257.4 |
| Cao (2022)^63^ | Left obstructive lesions, Coarctation | Syndromic [Ventriculomegaly] | LZTR1 (definitive) | c.243 T > G; c.a1206T > A | AD | NM_006767.4 |
| Slavotinek (2024)^60^ | Septal Defect, VSD, ASD | Syndromic | ROCK2 (limited) | c.2140C>T, (p.Arg714Ter) | De Novo, AD | NM_004850.4 |
| Slavotinek (2024)^60^ | Septal Defect, ASD | Syndromic | PRKD1 (limited) | c.2219G>A, p.Arg740Gln | De Novo, AD | NM_002742.3 |
| Slavotinek (2024)^60^ | Septal Defect, VSD, ASD | Isolated | MYH7 (limited) | c.1217_1218del, p.Val406fs | Maternal, AD | NM_000257.4 |
| Slavotinek (2024)^60^ | Septal Defect, ASD | Isolated | MYH6 (definitive) | c.4328C>A, p.Ala1443Asp | Maternal, AD | NM_002471.4 |
| Slavotinek (2024)^60^ | Single Ventricle, Single ventricle | Syndromic | CRELD1 (limited) | c.726G>T, p.Lys242Asn | AD | NM_001077415.3 |
| Slavotinek (2024)^60^ | Not specified | Syndromic | SOS1 (definitive) | c.1074+1966C>G | De Novo, AD | NM_005633.4 |
| **Author and year of publication** | **CHD type** | **Other findings** | **Gene (ClinGen Gene-Disease Validity Classification when available)** | **Variant** | **Inheritance** | **NM** |
| Hauser (2017)^57^ | Other, Ebstein's anomaly | Syndromic [Hydronephrosis, Wolff Parkinson White] | CITED2 | c.574A>G, p.Ser192Gly | Maternal | NM_006079.4 |
| Hauser (2017)^57^ | Conotruncal lesion, TGA | Syndromic [Hydronephrosis] | NODAL | c.397C>T, p.Gln133a | Maternal | NM_018055.4 |
| Hauser (2017)^57^ | Conotruncal lesion, TGA | Syndromic [Hydronephrosis] | CITED2 | c.A245G, p.His82Arg | Paternal | NM_006079.4 |
| Hauser (2017)^57^ | Other, Vascular ring | Isolated [Subglottic stenosis from  vascular ring] | GDF1 | c.G485A, p.Gly162Asp | Paternal | NM_001492.5 |
| Alankarage (2019)^58^ | Single ventricle | Isolated | INVS | c.646T>G, Trp216Gly | AD | NM_014425.4 |
| Alankarage (2019)^58^ | Septal Defect | Isolated | NOTCH1 | c.3122G>A, Gly1041Asp | AD | NM_017617.4 |
| Alankarage (2019)^58^ | Septal Defect | Isolated | CDK13 | c.2180C>T, Thr727Ile | AD | NM_003718.4 |
| Alankarage (2019)^58^ | Septal Defect | Isolated | FLNA | c.1451G>A, Arg484Gln | XR | NM_001456.3 |
| Alankarage (2019)^58^ | Septal Defect | Isolated | PRDM6 | c.1301A>T, Asp434Val | AD | NM_001136239.3 |
| Alankarage (2019)^58^ | Septal Defect | Isolated | NOTCH1 | c.2982C>G, Asn994Lys | AD | NM_017617.4 |
| Alankarage (2019)^58^ | Not specified | Isolated | FOXH1 | c.227G>A, Gly76Asp | AD | NM_003923.2 |
| Alankarage (2019)^58^ | Septal Defect | Isolated | FLNA | c.7534C>T, Arg2512Cys | XR | NM_001456.3 |
| Alankarage (2019)^58^ | Septal Defect | Isolated | NOTCH1 | c.5470C>T, Arg1824Trp | AD | NM_017617.4 |
| Alankarage (2019)^58^ | Not specified | Isolated | ZEB2 | c.1386G>T, Lys462Asn | AD | NM_014795.3 |
| Alankarage (2019)^58^ | Septal Defect | Syndromic | ELN | c.1150+1G>A, NA | AD | NM_000501.3 |
| Alankarage (2019)^58^ | Septal Defect | Syndromic | SON | c.3402T>G, Asp1134Glu | AD | NM_032195.2 |
| Alankarage (2019)^58^ | Not specified | Isolated | TBX5 | c.289A>G, Lys97Glu | AD | NM_000192.3 |
| Alankarage (2019)^58^ | Septal Defect | Syndromic | MYH1 | c.3560C>T, Thr1187Met | AD | NM_022844.2 |
| Alankarage (2019)^58^ | Not specified | Isolated | INVS | c.2432C>G, Pro811Arg | AD | NM_014425.4 |
| Alankarage (2019)^58^ | Not specified | Isolated | NOTCH1 | c.368C>T, Thr123Met | AD | NM_017617.4 |
| Alankarage (2019)^58^ | Not specified | Isolated | CHD7 | c.5533G>A, Gly1845Arg | AD | NM_017780.3 |
| Alankarage (2019)^58^ | Not specified | Isolated | ELN | c.326G>A, Gly109Asp | AD | NM_000501.3 |
| Alankarage (2019)^58^ | Not specified | Isolated | GATA5 | c.1178C>T, Ala393Val | AD | NM_080473.4 |
| Alankarage (2019)^58^ | Not specified | Syndromic | CHD4 | c.706G>A, Val236Met | AD | NM_001273.3 |
| Alankarage (2019)^58^ | Not specified | Syndromic | TGFBR2 | c.367A>T, Met123Leu | AD | NM_003242.5 |
| Alankarage (2019)^58^ | Not specified | Syndromic | INVS | c.3182dup, Asn1061Lysfs*20 | AD | NM_014425.4 |
| Alankarage (2019)^58^ | Septal Defect | Syndromic | ADAMTS10 | c.1117G>A, Glu373Lys | AD | NM_030957.3 |
| Alankarage (2019)^58^ | Not specified | Syndromic | MYH11 | c.5792C>A, Pro1931His | AD | NM_022844.2 |
| Alankarage (2019)^58^ | Not specified | Syndromic | NOTCH1 | c.1039G>A, Gly347Ser | AD | NM_017617.4 |
| Alankarage (2019)^58^ | Septal Defect | Syndromic | STRA6 | c.983G>T, Gly328Val | AD | NM_022369.3 |
| Alankarage (2019)^58^ | Septal Defect | Syndromic | ARID1A | c.3408G>A, Ala1136Ala | AD | NM_006015.5 |
| Alankarage (2019)^58^ | Not specified | Syndromic | FBN1 | c.4809A>G, Ile1603Met | AD | NM_000138.4 |
| Reuter (2020)^61^ | Heterotaxy, AVSD | Syndromic [Macrocephaly, left-sided liver, polysplenia, right-sided stomach, ultrastructural ciliary abnormalities] | DNAH9 | c.[4421A>G]; [13244T>C], p.[(Asp1474Gly)]; [(Ile4415Thr)] | Both Parents | (NM_001372.3) |
| Reuter (2020)^61^ | Heterotaxy, AVSD | Syndromic [Failure to thrive, short stature, borderline microcephaly, asplenia, right-sided stomach] | DNAH8 | c.[991A>G]; [10773C>G]`, p.[(Thr331Ala)]; [(Phe3591Leu)] | Maternal, Paternal | (NM_001206927.1) |
| Reuter (2020)^61^ | Conotruncal lesion, TOF | Isolated | TAB2 | c.1718C>T, p.(Thr573Ile) |  | NM_015093.4 |
| Reuter (2020)^61^ | PDA | Isolated | CHD7 | c.7031G>A, p.(Gly2344Asp) | Paternal | NM_017780.3 |
| Reuter (2020)^61^ | Conotruncal lesion, TOF | Isolated | CHD4 | c.28C>G, p.(Pro10Ala) | Paternal | NM_001273.2 |
| Reuter (2020)^61^ | Left obstructive lesions, HLHS | Isolated | GDF1 | c.1037C>T, p.(Ser346Phe) | Maternal | NM_001492.6 |
| Reuter (2020)^61^ | Other, PS, AI | Isolated | NOTCH1 | c.1934G>A, p.(Cys645Tyr) | Maternal | NM_017617.5 |
| Reuter (2020)^61^ | Conotruncal lesion, TOF | Syndromic [Congenital lymphedema] | FOXC2 | c.1339T>A, p.(Phe447Ile) | Paternal | NM_005251.3 |
| Reuter (2020)^61^ | Conotruncal lesion, TGA | Isolated | ZEB2 | c.3067+4T>C, p.? | De Novo | NM_014795.3 |
| Reuter (2020)^61^ | Left obstructive lesions, HLHS | Syndromic [Retrognathia, mild dysmorphisms] | SMAD6 | c.874+5G>A, p.? | Paternal | NM_005585.4 |
| Reuter (2020)^61^ | Left obstructive lesions, HLHS | Syndromic [ADHD, nephrocalcinosis, hypercalcemia, dysmorphisms (parietal bossing, broad forehead, preauricular pit)] | DNAH11 | c.[3494A>G]; [9952C>T], p.[(Asp1165Gly)]; [(Gln3318*)] | Paternal, Maternal | NM_001277115.1 |
| Reuter (2020)^61^ | Heterotaxy, unbalanced AVSD | Syndromic [Left-sided liver, polysplenia, superior mesenteric vein to the right of the superior mesenteric artery, stomach to the right, abnormal configuration of the colon (likely malrotation), macrocephaly, mild respiratory issues, ultrastructural ciliary abnormalities] | DNAH9 | c.[4421A>G];[13244T>C], p.[(Asp1474Gly)];[(Ile4415Thr)] | Paternal, Maternal | NM_001372.4 |
| Reuter (2020)^61^ | Heterotaxy | Syndromic [Short stature, failure to thrive, borderline microcephaly, right-sided liver extends across midline, asplenia, stomach to the right, right isomeric bronchi] | DNAH8 | c.[991A>G];[10773C>G], p.[(Thr331Ala)];[(Phe3591Leu)] | Paternal, Maternal | NM_001206927.1 |
| Reuter (2020)^61^ | Left obstructive lesions, Coarctation | Isolated | PRDM6 | c.742G>A, p.(Val248Met) | Paternal | NM_001136239.4 |
| Reuter (2020)^61^ | Left obstructive lesions, HLHS | Syndromic [Global developmental delay, bilateral cryptorchidism, strabismus, gastroesophageal reflux disease, distal tubular renal acidosis, hydronephrosis, short stature, failure to thrive, dysmorphisms] | DNAH1 | c.9049G>A, p.(Asp3017Asn) | Both Parents | NM_015512.4 |
| Reuter (2020)^61^ | Other, ccTGA | Syndromic [Speech delay, microcephaly, dysmorphisms] | ZFPM2 | c.1993G>A, p.(Gly665Arg) | Paternal | NM_012082.3 |
| Reuter (2020)^61^ | Conotruncal lesion, TOF | Isolated | KDR | c.1270G>A, p.(Gly424Ser) | Maternal | NM_002253.2 |
| Reuter (2020)^61^ | Left obstructive lesions, Coarctation | Syndromic [Minor dysmorphisms] | TBX1 | c.1309C>T, p.(Pro437Ser) | Maternal | NM_080647.1 |
| Reuter (2020)^61^ | Right obstructive lesions, PA/IVS | Syndromic [Specific learning disability, atonic seizures, stroke post cardiac surgery] | PLD1 | c.1276G>A, p.(Ala426Thr) | Maternal | NM_002662.5 |
| Reuter (2020)^61^ | Right obstructive lesions, TS/PS | Syndromic [Epicanthus, high forehead, depressed nasal bridge] | GATA5 | c.632A>G, p.(Asn211Ser) | Maternal | NM_080473.5 |
| Reuter (2020)^61^ | Conotruncal lesion, Truncus | Syndromic [Minor dysmorphisms] | ARID1A | c.1944C>A, p.(Asp648Glu) | Maternal | NM_006015.6 |
| Reuter (2020)^61^ | Left obstructive lesions, Coarctation | Isolated | TBX1 | c.*10+74G>A, p.? | Maternal | NM_005992.1 |
| Blue (2022)^59^ | Conotruncal lesion, TGA | Isolated | MYH11 | c.C4712T, p.A1571V |  | NM_002474 |
| Blue (2022)^59^ | Conotruncal lesion, TGA | Isolated | GATA5 | c.T698C, p.L233P |  | NM_080473 |
| Blue (2022)^59^ | Conotruncal lesion, TGA | Syndromic [multiple organ dysfunction syndrome] | SALL4 | c.A3101G, p.D1034G |  | NM_020436 |
| Blue (2022)^59^ | Conotruncal lesion, TGA | Isolated | FOXC1 | c.T1552A, p.L518M |  | NM_001453 |
| Blue (2022)^59^ | Conotruncal lesion, TGA | Isolated | DOCK6 | c.C2045T, p.P682L |  | NM_020812 |
| Blue (2022)^59^ | Conotruncal lesion, TGA | Isolated | ZFPM2 | c.2107A>C, p.Met703Leu |  | NM_012082.4 |
| Blue (2022)^59^ | Conotruncal lesion, TGA | Isolated | ZFPM2 | c.2107A>C, p.Met703Leu |  | NM_012082.4 |
| Cao (2022)^63^ | Venous abnormality, Venous abnormality | Syndromic [IUGR] | PKHD1 | c.2280-559G > A | AR | NM_138694.3 |
| Cao (2022)^63^ | Septal Defect, AVSD | Isolated | DNAH11 | c.3470 T > G, p.L1157R | AR | NM_001277115.2 |
| Munabi (2022)^62^ | Heterotaxy, hypoplastic RV | Syndromic [unilateral, left-sided cleft lip and cleft palate, right anophthalmia, central hypothyroidism, absent corpus callosum, and a right occipital skull defect with hydrocephaly at birth] | Apolipoprotein B (APOB) | c.12766G>A, p.Glu4256Lys | De novo | NM_000384.3 |
| Munabi (2022)^62^ | Heterotaxy, hypoplastic RV | Syndromic [unilateral, left-sided cleft lip and cleft palate, right  anophthalmia, central hypothyroidism, absent corpus callosum, and a  right occipital skull defect with hydrocephaly at birth] | Apolipoprotein  B (APOB) | c.11257T>C, p.Phe3753Leu |  | NM_000384.3 |
| Munabi (2022)^62^ | Conotruncal lesion, TOF | Syndromic [Bilateral complete cleft lip and palate. Other anomalies included global developmental delay and mildly dysplastic ears] | RNF213 | c.2213G>A, p.Ser738Asn | Possible De Novo | NM_020954.4 |
| Munabi (2022)^62^ | Conotruncal lesion, TOF | Syndromic [Bilateral complete cleft lip and palate. Other anomalies  included global developmental delay and mildly dysplastic ears] | SETX | c.7490G>A , c.1343A>G, p.Ser2497Asn, p.Asp448Gly | Possible De Novo | NM_001351527.2, NM_001351527.2 |
| Munabi (2022)^62^ | Conotruncal lesion, TOF | Syndromic [submucous CP at age 11. Other anomalies included right cryptorchidism, imperforate anus, hypoparathyroidism, and seizure disorder] | JAG1 | c.3560A>G, p.Asn1187Ser | Possible De Novo | NM_000214.3 |
| Slavotinek (2024)^60^ | PDA | Syndromic | DHX15 | c.1327A>G, p.(Lys443Glu) | De Novo, AD | NM_001358.3 |
| Slavotinek (2024)^60^ | Septal Defect, ASD | Syndromic | MACF1 | c.5464C>T , (p.Gln1822Ter) | AD | NM_012090.5 |
| Slavotinek (2024)^60^ | Septal Defect, VSD, ASD | Syndromic | MAPK8IP3 | c.2429C>A, (p.Ala810Asp) | AD | NM_001040439.1 |
| Slavotinek (2024)^60^ | Septal Defect, ASD | Syndromic | TRAF7 | c.1510G>C, p.Asp504His | De Novo, AD | NM_032271.3 |
| Slavotinek (2024)^60^ | Septal Defect, ASD | Syndromic | CARM1 | c.362A>T, p.Tyr121Phe | De Novo, AD | NM_199141.2 |
| Slavotinek (2024)^60^ | Septal Defect, ASD | Syndromic | ADAM23 | c.2268del, p.Cys757fs | De Novo, AD | NM_003812.4 |
| Slavotinek (2024)^60^ | Septal Defect, ASD | Syndromic | PTPRM | c.1749A>G, p.Ile583Met | AD | NM_002845.3 |
| Slavotinek (2024)^60^ | Septal Defect, ASD | Isolated | TOR1A | c.949A>T, p.Lys317Ter | AD | NM_000113.2 |
| Slavotinek (2024)^60^ | Other, ASD, PPS | Syndromic | ALG12 | c.319G>A, p.Val107Met | AD | NM_024105.4 |
| Slavotinek (2024)^60^ | Other, ASD, PPS | Syndromic | ALG12 | c.470-3C>T | AD | NM_024105.4 |
| Slavotinek (2024)^60^ | Other, ASD, PPS | Syndromic | CD96 | c.934del, p.Asp312fs | AD | NM_005816.5 |
| Slavotinek (2024)^60^ | Septal Defect, VSD, ASD | Syndromic | NIPBL | c.6705_6707del, p.Lys2235del |  | NM_133433.4 |
| Slavotinek (2024)^60^ | PDA | Isolated | TMEM260 | c.377C>T , p.Ala126Val | Both Parents, AR | NM_017799.4 |
| Slavotinek (2024)^60^ | PDA | Syndromic | CSNK1E | c.287G>T, p.Cys96Phe | De Novo, X-linked | NM_152221.3 |
| Slavotinek (2024)^60^ | PDA | Syndromic | RINT1 | c.1285C>A , p.Leu429Ile | Maternal, X-linked | NM_021930.6 |
| Slavotinek (2024)^60^ | Septal Defect, VSD, ASD | Syndromic | U2AF2 | c.791G>A, p.Gly264Glu | Paternal | NM_007279.3 |
| Slavotinek (2024)^60^ | Left obstructive lesions, Coarctation | Syndromic | SMARCA1 | c.1874A>G, p.Asp625Gly | Maternal | NM_001282874.2 |
| Slavotinek (2024)^60^ | Left obstructive lesions, Coarctation | Syndromic | SMARCA1 | c.2354A>G, p.Asn785Ser | Maternal | NM_001282874.2 |
| Slavotinek (2024)^60^ | Left obstructive lesions, Coarctation | Syndromic | SAMD4B | c.1569G>C, p.Trp523Cys | Maternal | NM_018028.3 |
| Slavotinek (2024)^60^ | Septal Defect, VSD, ASD | Syndromic | USP9X | c.4817A>G, p.Asp1606Gly | De Novo, AD | NM_001039591.3 |
| Slavotinek (2024)^60^ | Conotruncal lesion, TGA, PA | Syndromic | KMT2D | c.15989C>G, p.Pro5330Arg | Maternal, AD | NM_003482.3 |
| Slavotinek (2024)^60^ | Septal Defect, ASD | Syndromic | NUP214 | c.1412C>T, p.Ser471Phe | AR | NM_005085.4 |
| Slavotinek (2024)^60^ | Septal Defect, ASD | Syndromic | NUP214 | c.3344C>T, p.Thr1115Met | AR | NM_005085.4 |
| Slavotinek (2024)^60^ | Septal Defect, VSD, ASD | Syndromic | HCFC1 | c.6069G>T, p.Met2023Ile | Maternal, AR | NM_005334.3 |
| Slavotinek (2024)^60^ | Other, PPS | Syndromic | CDH7 | c.2096G>A, p.Ser699Asn | Paternal, AD | NM_017780.4 |
| Slavotinek (2024)^60^ | Septal Defect, VSD, ASD | Syndromic | MID1 | c.1495G>A, p.Val499Met | Maternal, XR | NM_000381.4 |
| Slavotinek (2024)^60^ | Conotruncal lesion, TOF | Syndromic | SMARCA4 | c.2476G>T, p.Ala826Ser | AD | NM_003072.5 |
| Slavotinek (2024)^60^ | Septal Defect, ASD | Syndromic | ERCC2 | c.679C>T, p.Arg227Cys | AR | NM_000400.3 |
| Slavotinek (2024)^60^ | Septal Defect, ASD | Syndromic | ERCC2 | c.462C>G, p.His154Gln | AR | NM_000400.3 |
| Slavotinek (2024)^60^ | Septal Defect, ASD | Syndromic | UPS11 | c.2420G>A, p.Arg807Gln | Maternal, AR | NM_001371072.1 |
| Slavotinek (2024)^60^ | Septal Defect, ASD | Syndromic | DHCR7 | c.99-16G>A | AR | NM_001360.3 |
| Slavotinek (2024)^60^ | Septal Defect, ASD | Syndromic | DHCR7 | c.-146C>T | AR | NM_001360.3 |
| Slavotinek (2024)^60^ | Septal Defect, VSD, ASD | Syndromic | PREPL | c.1244T>C, p.Leu415Ser | Both Parents, AR | NM_001171613.2 |
| Slavotinek (2024)^60^ | PDA | Syndromic | SYNE1 | c.26152A>G, p.Arg8718Gly | Both Parents, AR | NM_182961.4 |
| ADHD: Attention Deficit Hyperactivity Disorder, AI: Aortic Insufficiency, ASD: Atrial Septal Defect, AVSD: Atrioventricular Septal Defect, ccTGA: Congenitally Corrected Transposition of the Great Arteries, CHD: Congenital Heart Disease, CP: Cleft Palate, HLHS: Hypoplastic Left Heart Syndrome, IUGR: Intrauterine Growth Restriction, IVS: Intact Ventricular Septum, NM: Nomenclature, OCD: Obsessive-Compulsive Disorder, PA: Pulmonary Atresia, PA/IVS: Pulmonary Atresia with Intact Ventricular Septum, PDA: Patent Ductus Arteriosus, PPS: Peripheral Pulmonary Stenosis, PS: Pulmonary Stenosis, RV: Right Ventricle, TGA: Transposition of the Great Arteries, TOF: Tetralogy of Fallot, TS: Tricuspid Stenosis, VSD: Ventricular Septal Defect | | | | | | |

1. Rinné S, Stallmeyer B, Pinggera A, et al. Whole Exome Sequencing Identifies a Heterozygous Variant in the Cav1.3 Gene CACNA1D Associated with Familial Sinus Node Dysfunction and Focal Idiopathic Epilepsy. *IJMS*. 2022;23(22):14215. doi:10.3390/ijms232214215

2. Calpena E, Hervieu A, Kaserer T, et al. De Novo Missense Substitutions in the Gene Encoding CDK8, a Regulator of the Mediator Complex, Cause a Syndromic Developmental Disorder. *The American Journal of Human Genetics*. 2019;104(4):709-720. doi:10.1016/j.ajhg.2019.02.006

3. Zhao Y, Wang Y, Shi L, et al. Chromatin regulators in the TBX1 network confer risk for conotruncal heart defects in 22q11.2DS. *npj Genom Med*. 2023;8(1):17. doi:10.1038/s41525-023-00363-y

4. Baker EK, Solivio B, Pode‐Shakked B, et al. *PPP2R1A* neurodevelopmental disorder is associated with congenital heart defects. *American J of Med Genetics Pt A*. 2022;188(11):3262-3277. doi:10.1002/ajmg.a.62946

5. Zhu N, Swietlik EM, Welch CL, et al. Rare variant analysis of 4241 pulmonary arterial hypertension cases from an international consortium implicates FBLN2, PDGFD, and rare de novo variants in PAH. *Genome Med*. 2021;13(1):80. doi:10.1186/s13073-021-00891-1

6. Miller DB, Piccolo SR. A Survey of Compound Heterozygous Variants in Pediatric Cancers and Structural Birth Defects. *Front Genet*. 2021;12:640242. doi:10.3389/fgene.2021.640242

7. Theis JL, Vogler G, Missinato MA, et al. Patient-specific genomics and cross-species functional analysis implicate LRP2 in hypoplastic left heart syndrome. *eLife*. 2020;9:e59554. doi:10.7554/eLife.59554

8. Manshaei R, Merico D, Reuter MS, et al. Genes and Pathways Implicated in Tetralogy of Fallot Revealed by Ultra-Rare Variant Burden Analysis in 231 Genome Sequences. *Front Genet*. 2020;11:957. doi:10.3389/fgene.2020.00957

9. Birker K, Ge S, Kirkland NJ, et al. Mitochondrial MICOS complex genes, implicated in hypoplastic left heart syndrome, maintain cardiac contractility and actomyosin integrity. *eLife*. 2023;12:e83385. doi:10.7554/eLife.83385

10. Liu Y, Chang X, Glessner J, et al. Association of Rare Recurrent Copy Number Variants With Congenital Heart Defects Based on Next-Generation Sequencing Data From Family Trios. *Front Genet*. 2019;10:819. doi:10.3389/fgene.2019.00819

11. Richter F, Morton SU, Kim SW, et al. Genomic analyses implicate noncoding de novo variants in congenital heart disease. *Nat Genet*. 2020;52(8):769-777. doi:10.1038/s41588-020-0652-z

12. Stittrich AB, Lehman A, Bodian DL, et al. Mutations in NOTCH1 Cause Adams-Oliver Syndrome. *The American Journal of Human Genetics*. 2014;95(3):275-284. doi:10.1016/j.ajhg.2014.07.011

13. Theis JL, Shatila SDH, Fogarty ZC, Bamlet WR, Olson TM. Genome-Wide Association and Inheritance-Based Analyses Implicate Unconventional Myosin Genes in Hypoplastic Left Heart Syndrome. *Circ: Genomic and Precision Medicine*. 2023;16(1). doi:10.1161/CIRCGEN.122.003761

14. Theis JL, Niaz T, Sundsbak RS, et al. *CELSR1* Risk Alleles in Familial Bicuspid Aortic Valve and Hypoplastic Left Heart Syndrome. *Circ: Genomic and Precision Medicine*. 2022;15(2). doi:10.1161/CIRCGEN.121.003523

15. Theis JL, Hu JJ, Sundsbak RS, et al. Genetic Association Between Hypoplastic Left Heart Syndrome and Cardiomyopathies. *Circ: Genomic and Precision Medicine*. 2021;14(1):e003126. doi:10.1161/CIRCGEN.120.003126

16. Wang C, Lv H, Ling X, et al. Association of assisted reproductive technology, germline de novo mutations and congenital heart defects in a prospective birth cohort study. *Cell Res*. 2021;31(8):919-928. doi:10.1038/s41422-021-00521-w

17. Grunert M, Appelt S, Grossfeld P, Sperling SR. The Needle in the Haystack—Searching for Genetic and Epigenetic Differences in Monozygotic Twins Discordant for Tetralogy of Fallot. *JCDD*. 2020;7(4):55. doi:10.3390/jcdd7040055

18. Trevino CE, Holleman AM, Corbitt H, et al. Identifying genetic factors that contribute to the increased risk of congenital heart defects in infants with Down syndrome. *Sci Rep*. 2020;10(1):18051. doi:10.1038/s41598-020-74650-4

19. Theis JL, Zimmermann MT, Evans JM, et al. Recessive *MYH6* Mutations in Hypoplastic Left Heart With Reduced Ejection Fraction. *Circ Cardiovasc Genet*. 2015;8(4):564-571. doi:10.1161/CIRCGENETICS.115.001070

20. Wang Y, Du X, Zhou Z, et al. A gain-of-function ACTC1 3′UTR mutation that introduces a miR-139-5p target site may be associated with a dominant familial atrial septal defect. *Sci Rep*. 2016;6(1):25404. doi:10.1038/srep25404

21. Robinson HK, Zaklyazminskaya E, Povolotskaya I, et al. Biallelic variants in *PPP1R13L* cause paediatric dilated cardiomyopathy. *Clinical Genetics*. 2020;98(4):331-340. doi:10.1111/cge.13812

22. Cheng H, Dharmadhikari AV, Varland S, et al. Truncating Variants in NAA15 Are Associated with Variable Levels of Intellectual Disability, Autism Spectrum Disorder, and Congenital Anomalies. *The American Journal of Human Genetics*. 2018;102(5):985-994. doi:10.1016/j.ajhg.2018.03.004

23. Gu X, Han L, Chen J, et al. Antenatal screening and diagnosis of tuberous sclerosis complex by fetal echocardiography and targeted genomic sequencing. *Medicine*. 2018;97(15):e0112. doi:10.1097/MD.0000000000010112

24. Johnston JJ, Van Der Smagt JJ, Rosenfeld JA, et al. Autosomal recessive Noonan syndrome associated with biallelic LZTR1 variants. *Genetics in Medicine*. 2018;20(10):1175-1185. doi:10.1038/gim.2017.249

25. Bjornsson T, Thorolfsdottir RB, Sveinbjornsson G, et al. A rare missense mutation in *MYH6* associates with non-syndromic coarctation of the aorta. *European Heart Journal*. 2018;39(34):3243-3249. doi:10.1093/eurheartj/ehy142

26. Reuter MS, Jobling R, Chaturvedi RR, et al. Haploinsufficiency of vascular endothelial growth factor related signaling genes is associated with tetralogy of Fallot. *Genetics in Medicine*. 2019;21(4):1001-1007. doi:10.1038/s41436-018-0260-9

27. Qiao XH, Wang Q, Wang J, et al. A novel NR2F2 loss-of-function mutation predisposes to congenital heart defect. *European Journal of Medical Genetics*. 2018;61(4):197-203. doi:10.1016/j.ejmg.2017.12.003

28. Da Costa SS, Fishman V, Pinheiro M, et al. A germline chimeric KANK1-DMRT1 transcript derived from a complex structural variant is associated with a congenital heart defect segregating across five generations. *Chromosome Res*. 2024;32(2):6. doi:10.1007/s10577-024-09750-2

29. Allred ET, Perens EA, Coufal NG, Sanford Kobayashi E, Kingsmore SF, Dimmock DP. Genomic sequencing has a high diagnostic yield in children with congenital anomalies of the heart and urinary system. *Front Pediatr*. 2023;11:1157630. doi:10.3389/fped.2023.1157630

30. Helm BM, Ware SM. Clinical Decision Analysis of Genetic Evaluation and Testing in 1013 Intensive Care Unit Infants with Congenital Heart Defects Supports Universal Genetic Testing. *Genes*. 2024;15(4):505. doi:10.3390/genes15040505

31. Wu B, Kang W, Wang Y, et al. Application of Full-Spectrum Rapid Clinical Genome Sequencing Improves Diagnostic Rate and Clinical Outcomes in Critically Ill Infants in the China Neonatal Genomes Project*. *Critical Care Medicine*. 2021;49(10):1674-1683. doi:10.1097/CCM.0000000000005052

32. Owen MJ, Wright MS, Batalov S, et al. Reclassification of the Etiology of Infant Mortality With Whole-Genome Sequencing. *JAMA Netw Open*. 2023;6(2):e2254069. doi:10.1001/jamanetworkopen.2022.54069

33. Chen W, Zhang Y, Yang S, et al. Bi-Allelic Mutations in *NUP205* and *NUP210* Are Associated With Abnormal Cardiac Left-Right Patterning. *Circ: Genomic and Precision Medicine*. 2019;12(7):e002492. doi:10.1161/CIRCGEN.119.002492

34. Chang Y, Wacker J, Ingles J, et al. *TBX20* loss-of-function variants in families with left ventricular non-compaction cardiomyopathy. *J Med Genet*. 2024;61(2):171-175. doi:10.1136/jmg-2023-109455

35. Rosenhahn E, O’Brien TJ, Zaki MS, et al. Bi-allelic loss-of-function variants in PPFIBP1 cause a neurodevelopmental disorder with microcephaly, epilepsy, and periventricular calcifications. *The American Journal of Human Genetics*. 2022;109(8):1421-1435. doi:10.1016/j.ajhg.2022.06.008

36. Du H, Jolly A, Grochowski CM, et al. The multiple de novo copy number variant (MdnCNV) phenomenon presents with peri-zygotic DNA mutational signatures and multilocus pathogenic variation. *Genome Med*. 2022;14(1):122. doi:10.1186/s13073-022-01123-w

37. Chan KCA, Jiang P, Sun K, et al. Second generation noninvasive fetal genome analysis reveals de novo mutations, single-base parental inheritance, and preferred DNA ends. *Proc Natl Acad Sci USA*. 2016;113(50). doi:10.1073/pnas.1615800113

38. Holt RJ, Young RM, Crespo B, et al. De Novo Missense Variants in FBXW11 Cause Diverse Developmental Phenotypes Including Brain, Eye, and Digit Anomalies. *The American Journal of Human Genetics*. 2019;105(3):640-657. doi:10.1016/j.ajhg.2019.07.005

39. Tripolszki K, Sasaki E, Hotakainen R, et al. An X‐linked syndrome with severe neurodevelopmental delay, hydrocephalus, and early lethality caused by a missense variation in the *OTUD5* gene. *Clinical Genetics*. 2021;99(2):303-308. doi:10.1111/cge.13873

40. Goos JAC, Swagemakers SMA, Twigg SRF, et al. Identification of causative variants in TXNL4A in Burn-McKeown syndrome and isolated choanal atresia. *Eur J Hum Genet*. 2017;25(10):1126-1133. doi:10.1038/ejhg.2017.107

41. Zhou Z, Huang X, Tang X, et al. Heterozygous nonsense variants in laminin subunit 3α resulting in Ebstein’s anomaly. *Human Genetics and Genomics Advances*. 2023;4(4):100227. doi:10.1016/j.xhgg.2023.100227

42. Miceikaite I, Fagerberg C, Brasch‐Andersen C, et al. Comprehensive prenatal diagnostics: Exome versus genome sequencing. *Prenatal Diagnosis*. 2023;43(9):1132-1141. doi:10.1002/pd.6402

43. Acevedo JM, Lee S, Gotteiner N, Lay AS, Patel A. Total anomalous pulmonary venous connection ( TAPVC ): A familial cluster of 3 siblings. *Echocardiography*. 2017;34(10):1531-1535. doi:10.1111/echo.13665

44. Hildebrandt CC, Patel N, Graham JM, et al. Further delineation of van den Ende‐Gupta syndrome: Genetic heterogeneity and overlap with congenital heart defects and skeletal malformations syndrome. *American J of Med Genetics Pt A*. 2021;185(7):2136-2149. doi:10.1002/ajmg.a.62194

45. Chung JH, Cai J, Suskin BG, Zhang Z, Coleman K, Morrow BE. Whole-Genome Sequencing and Integrative Genomic Analysis Approach on Two 22q11.2 Deletion Syndrome Family Trios for Genotype to Phenotype Correlations. *Human Mutation*. 2015;36(8):797-807. doi:10.1002/humu.22814

46. Nash D, Arrington CB, Kennedy BJ, et al. Shared Segment Analysis and Next-Generation Sequencing Implicates the Retinoic Acid Signaling Pathway in Total Anomalous Pulmonary Venous Return (TAPVR). Schubert M, ed. *PLoS ONE*. 2015;10(6):e0131514. doi:10.1371/journal.pone.0131514

47. Hrstka SCL, Li X, Nelson TJ, Wanek Program Genetics Pipeline Group. *NOTCH1* -Dependent Nitric Oxide Signaling Deficiency in Hypoplastic Left Heart Syndrome Revealed Through Patient-Specific Phenotypes Detected in Bioengineered Cardiogenesis. *Stem Cells*. 2017;35(4):1106-1119. doi:10.1002/stem.2582

48. Xiao F, Zhang X, Morton SU, et al. Functional dissection of human cardiac enhancers and noncoding de novo variants in congenital heart disease. *Nat Genet*. 2024;56(3):420-430. doi:10.1038/s41588-024-01669-y

49. Theis JL, Hrstka SCL, Evans JM, et al. Compound heterozygous NOTCH1 mutations underlie impaired cardiogenesis in a patient with hypoplastic left heart syndrome. *Hum Genet*. 2015;134(9):1003-1011. doi:10.1007/s00439-015-1582-1

50. Tan M, Wang X, Liu H, et al. Genetic Diagnostic Yield and Novel Causal Genes of Congenital Heart Disease. *Front Genet*. 2022;13:941364. doi:10.3389/fgene.2022.941364

51. Zhou J, Yang Z, Sun J, et al. Whole Genome Sequencing in the Evaluation of Fetal Structural Anomalies: A Parallel Test with Chromosomal Microarray Plus Whole Exome Sequencing. *Genes*. 2021;12(3):376. doi:10.3390/genes12030376

52. Xu J, Wu Q, Wang L, et al. Next-generation sequencing identified genetic.

53. Wang Y, Du X, Zhou Z, et al. A gain-of-function ACTC1 3′UTR mutation that introduces a miR-139-5p target site may be associated with a dominant familial atrial septal defect. *Sci Rep*. 2016;6(1):25404. doi:10.1038/srep25404

54. Jia Y, Chen J, Zhong J, et al. Novel rare mutation in a conserved site of *PTPRB* causes human hypoplastic left heart syndrome. *Clinical Genetics*. 2023;103(1):79-86. doi:10.1111/cge.14234

55. GU X. Prenatal echocardiographic diagnosis and genetic analysis of fetal atrioventricular septal defect. *Chinese Journal of Medical Imaging Technology*. Published online 2020:918-922.

56. Sweeney NM, Nahas SA, Chowdhury S, et al. Author Correction: Rapid whole genome sequencing impacts care and resource utilization in infants with congenital heart disease. *npj Genom Med*. 2021;6(1):38. doi:10.1038/s41525-021-00205-9

57. Hauser NS, Solomon BD, Vilboux T, Khromykh A, Baveja R, Bodian DL. Experience with genomic sequencing in pediatric patients with congenital cardiac defects in a large community hospital. *Molec Gen & Gen Med*. 2018;6(2):200-212. doi:10.1002/mgg3.357

58. Alankarage D, Ip E, Szot JO, et al. Identification of clinically actionable variants from genome sequencing of families with congenital heart disease. *Genetics in Medicine*. 2019;21(5):1111-1120. doi:10.1038/s41436-018-0296-x

59. Blue GM, Mekel M, Das D, et al. Whole genome sequencing in transposition of the great arteries and associations with clinically relevant heart, brain and laterality genes. *American Heart Journal*. 2022;244:1-13. doi:10.1016/j.ahj.2021.10.185

60. Slavotinek AM, Thompson ML, Martin LJ, Gelb BD. Diagnostic yield after next-generation sequencing in pediatric cardiovascular disease. *Human Genetics and Genomics Advances*. 2024;5(3):100286. doi:10.1016/j.xhgg.2024.100286

61. Reuter MS, Chaturvedi RR, Liston E, et al. The Cardiac Genome Clinic: implementing genome sequencing in pediatric heart disease. *Genetics in Medicine*. 2020;22(6):1015-1024. doi:10.1038/s41436-020-0757-x

62. Munabi NCO, Mikhail S, Toubat O, et al. High prevalence of deleterious mutations in concomitant nonsyndromic cleft and outflow tract heart defects. *American J of Med Genetics Pt A*. 2022;188(7):2082-2095. doi:10.1002/ajmg.a.62748

63. Cao Y, Chau MHK, Zheng Y, et al. Exploring the diagnostic utility of genome sequencing for fetal congenital heart defects. *Prenatal Diagnosis*. 2022;42(7):862-872. doi:10.1002/pd.6151
