# Supplementary figures and images for: Yield of Whole Genome Sequencing for Pathogenic Single Nucleotide Variants in Congenital Heart Disease: A Systematic Review and Meta‐Analysis
Source: Prenat Diagn. 2025 Sep 4;46(5-6):780–818. doi: 10.1002/pd.6878 (PMC13170038; doi:10.1002/pd.6878)

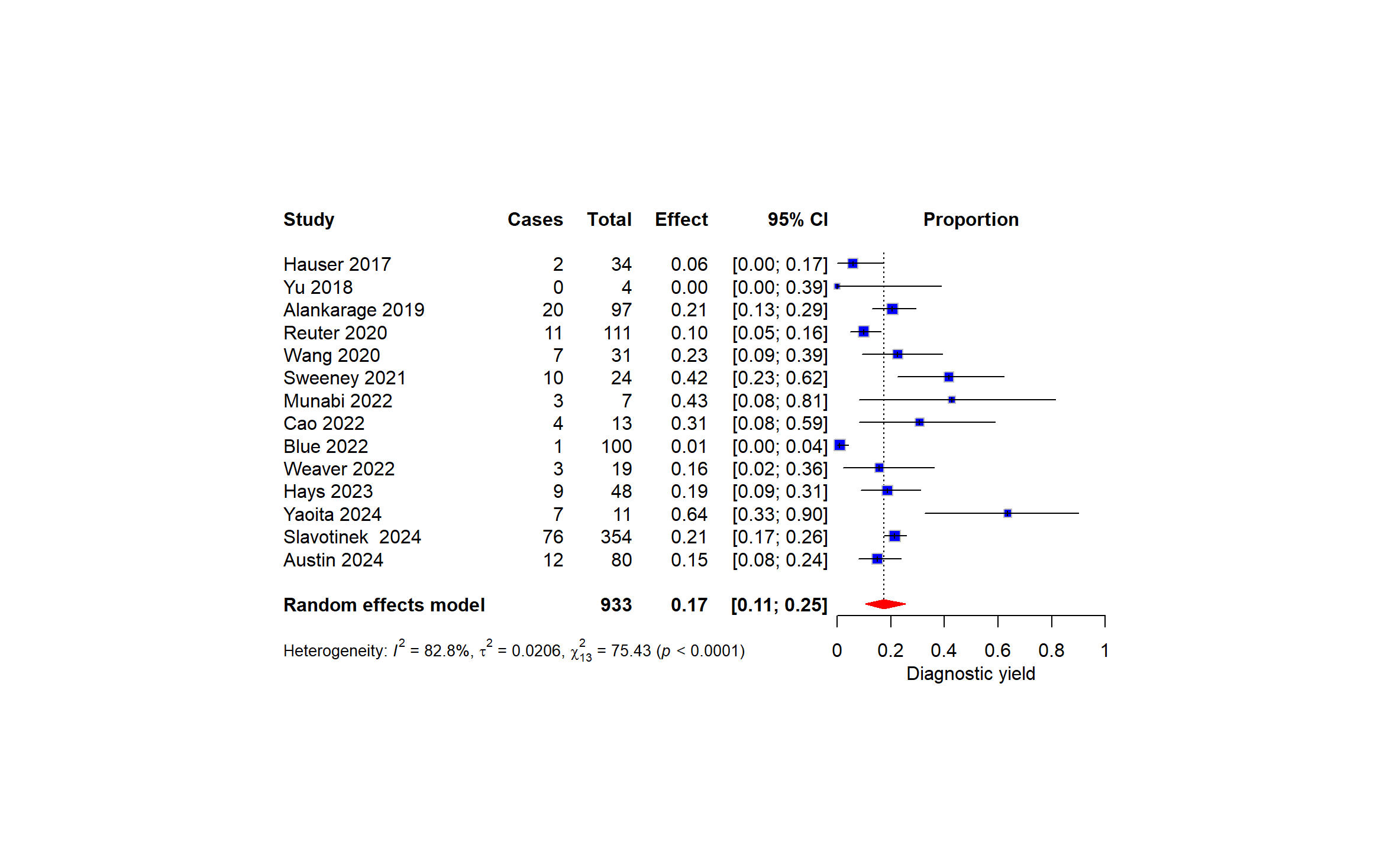

Supplement: Supplementary file 2 — Figure S1: Yield of whole genome sequencing in all CHD cases. [file PD-46-780-s006.jpg]

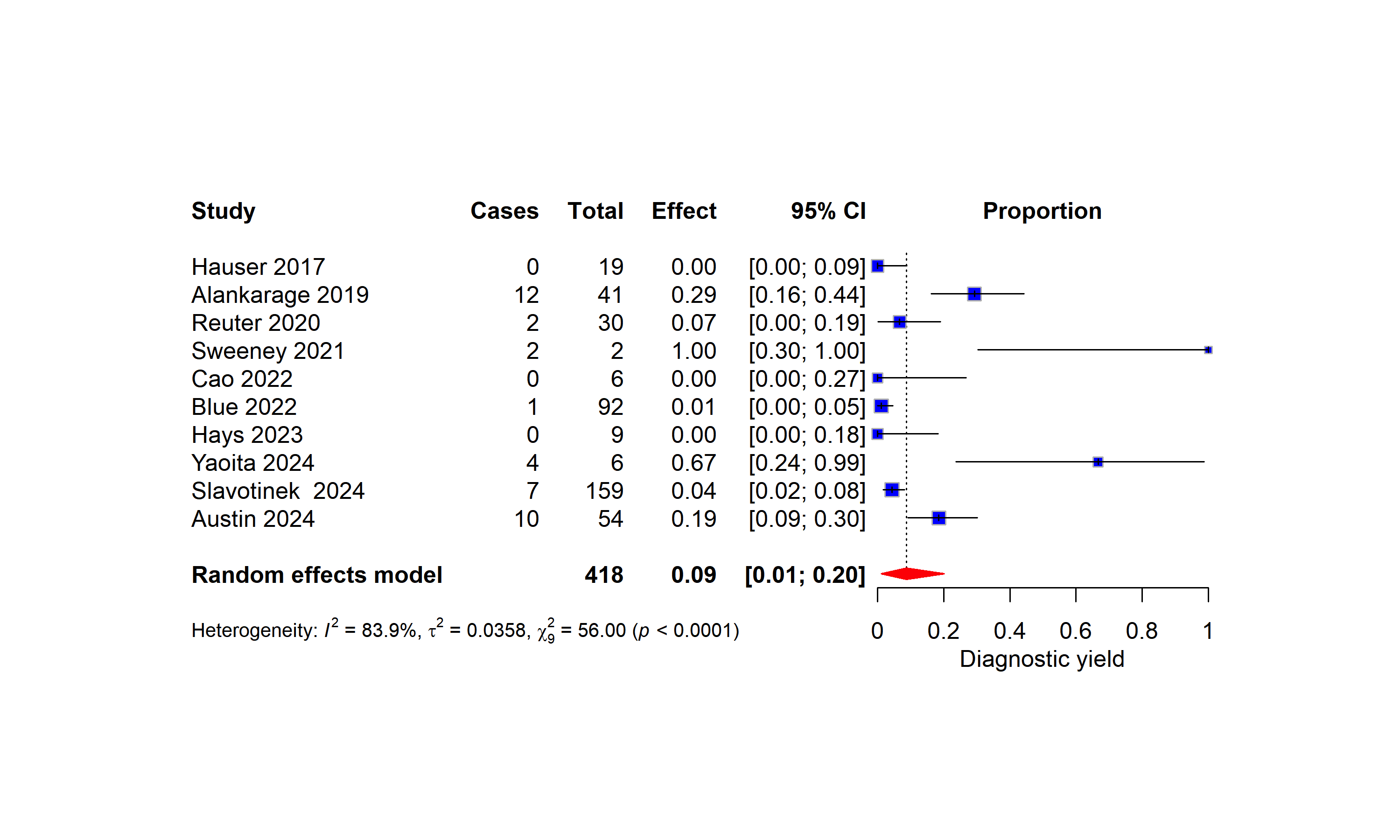

Supplement: Supplementary file 3 — Figure S2: Yield of whole genome sequencing in isolated CHD cases. [file PD-46-780-s002.jpg]

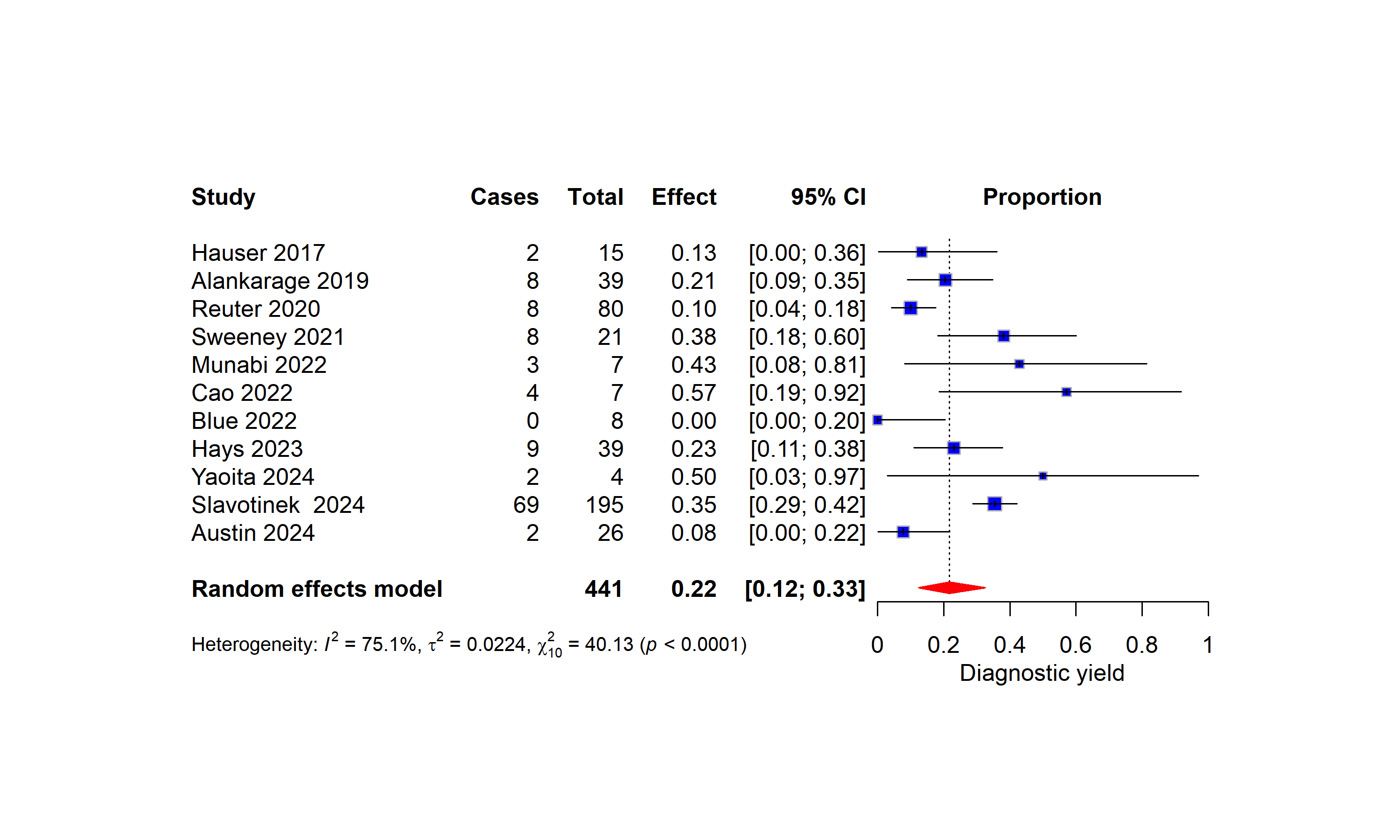

Supplement: Supplementary file 4 — Figure S3: Yield of whole genome sequencing in CHD with extracardiac abnormalities not accounting for developmental abnormalities. [file PD-46-780-s003.jpg]

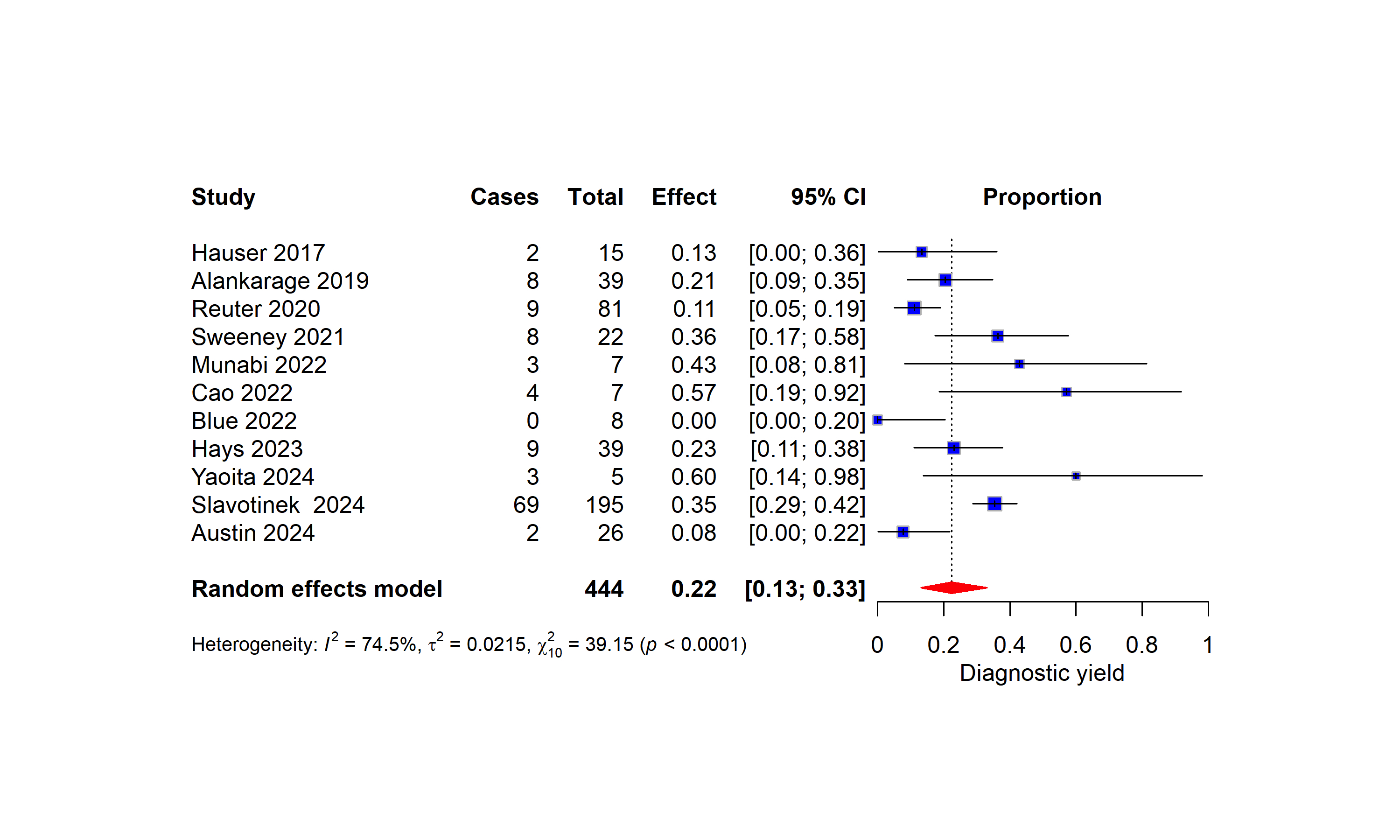

Supplement: Supplementary file 5 — Figure S4: Yield of whole genome sequencing in syndromic CHD cases. [file PD-46-780-s001.jpg]

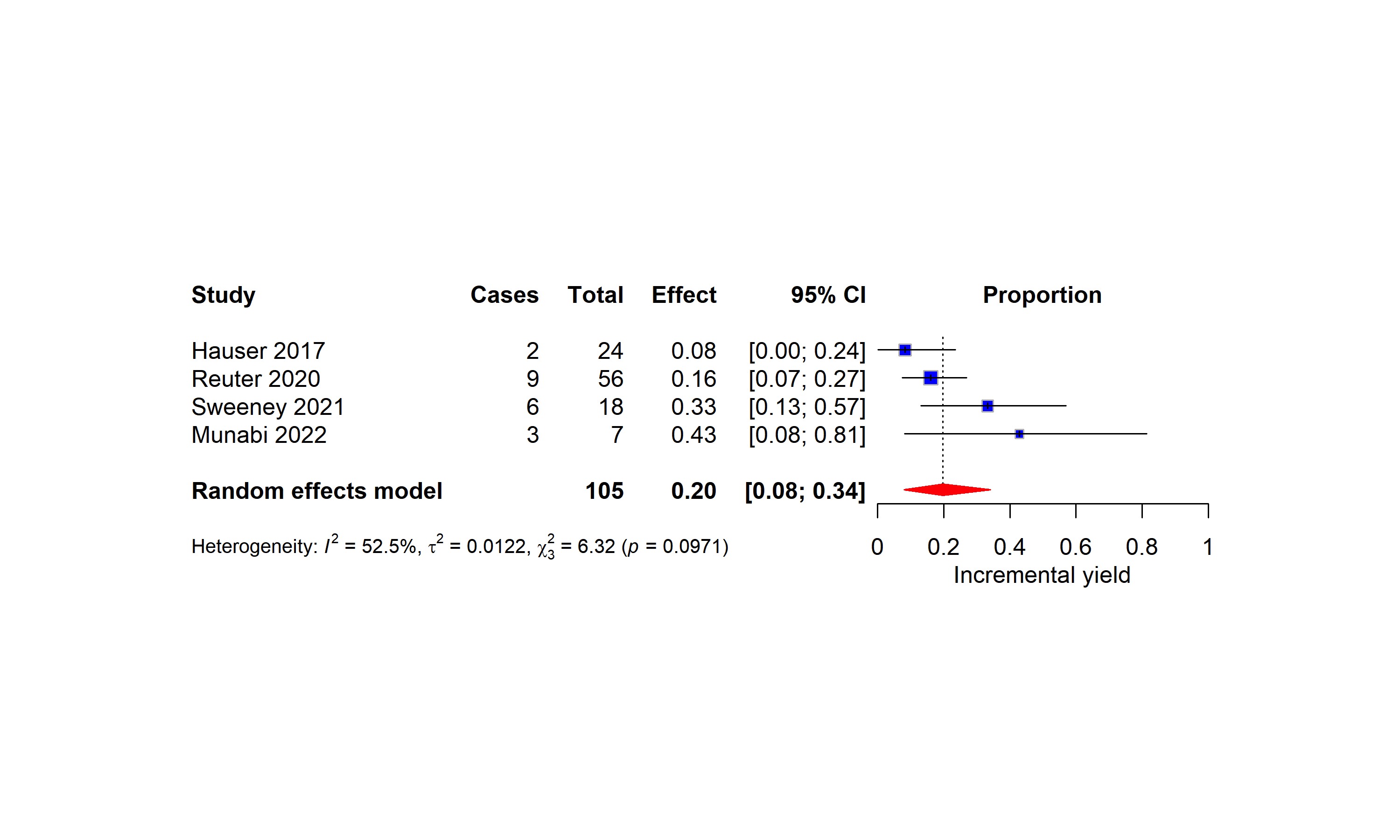

Supplement: Supplementary file 6 — Figure S5: Incremental yield of whole genome sequencing over CMA. [file PD-46-780-s005.jpg]
